# G protein-coupled receptors in neurodegenerative diseases and psychiatric disorders

**DOI:** 10.1038/s41392-023-01427-2

**Published:** 2023-05-03

**Authors:** Thian-Sze Wong, Guangzhi Li, Shiliang Li, Wei Gao, Geng Chen, Shiyi Gan, Manzhan Zhang, Honglin Li, Song Wu, Yang Du

**Affiliations:** 1 Kobilka Institute of Innovative Drug Discovery, Shenzhen Key Laboratory of Steroid Drug Discovery and Development, School of Medicine, The Chinese University of Hong Kong, 518172 Shenzhen, Guangdong China; 2 School of Medicine, Tsinghua University, 100084 Beijing, China; 3 Institute of Urology, The Affiliated Luohu Hospital of Shenzhen University, Shenzhen University, 518000 Shenzhen, Guangdong China; 4 Shanghai Key Laboratory of New Drug Design, School of Pharmacy, East China University of Science and Technology, 200237 Shanghai, China; 5 Innovation Center for AI and Drug Discovery, East China Normal University, 200062 Shanghai, China; 6 Department of Urology, South China Hospital, Health Science Center, Shenzhen University, 518116 Shenzhen, Guangdong China

**Keywords:** Neuroscience, Diseases of the nervous system

## Abstract

Neuropsychiatric disorders are multifactorial disorders with diverse aetiological factors. Identifying treatment targets is challenging because the diseases are resulting from heterogeneous biological, genetic, and environmental factors. Nevertheless, the increasing understanding of G protein-coupled receptor (GPCR) opens a new possibility in drug discovery. Harnessing our knowledge of molecular mechanisms and structural information of GPCRs will be advantageous for developing effective drugs. This review provides an overview of the role of GPCRs in various neurodegenerative and psychiatric diseases. Besides, we highlight the emerging opportunities of novel GPCR targets and address recent progress in GPCR drug development.

## Introduction

**Fig. 1:**
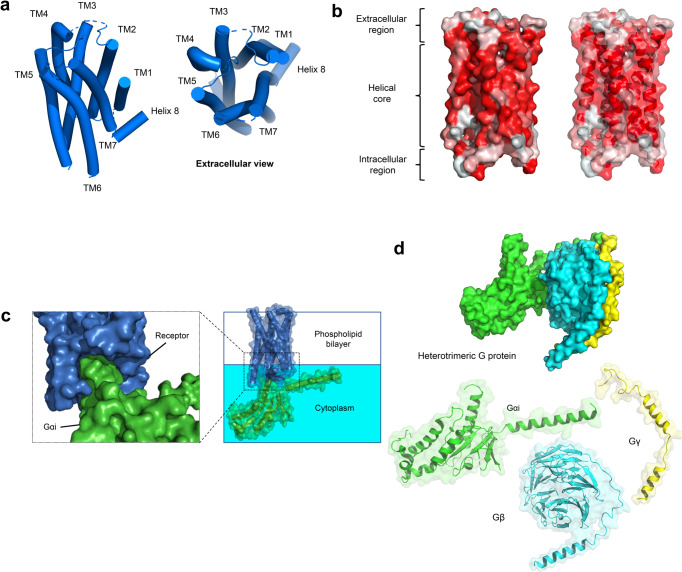
Structure features of active GPCR. **a** Orthosteric pocket forms by the helical core of 5-HT2A receptor (marine blue, PDB 6WHA). **b** Solvent-accessible surface. Hydrophobic surface (red); hydrophilic surface (white). **c** Activated GPCR opens cytosolic pocket for G protein coupling. **d** Heterotrimeric G protein, Monomeric Gαi, Gβγ. **d** Activated 5-HT2A receptor forms a cytoplasmic pocket which allows G-protein coupling

The nervous system employs membrane receptors to detect extracellular stimuli and transmit signals across the cell membrane. As the largest membrane protein family, G protein-coupled receptors (GPCRs) allow the nervous system to respond accurately to external stimuli and internal states. GPCRs are structurally similar transmembrane proteins containing seven transmembrane (TM) α-helices linked by three extracellular loops and three intracellular loops.^1^ The unique ligand binding pockets formed by the 7TM regions allow the receptor to engage with various stimuli, including neurotransmitters, nucleotides, amines, peptides, cytokines, and hormones in the extracellular environment (Fig. 1).^2^ Through expressing GPCRs with different ligand-recognizing abilities, the nervous system could filter and select particular signals to respond.^3^ Furthermore, the intrinsic ligand selectivity of neuronal GPCRs allows crosstalk and proper integration between signal transduction pathways. GPCRs drive signal transduction via two major modulators: heterotrimeric G protein and arrestins. Characterizing the physiological functions of GPCRs in the nervous system and pathological mechanisms in disease models could accelerate GPCR-targeted drug development.

The progressive dysfunction of neural tissues in the central and peripheral nervous systems is the hallmark feature of neurodegenerative diseases. Neurodegenerative diseases are increasing in the elderly population.^4^ It is estimated that neurodegenerative diseases affect over 50 million people across the globe.^5^ Alzheimer’s disease, Huntington’s disease, Parkinson’s disease, and Multiple sclerosis are representative examples. Currently, there is no effective cure. The pathogenesis and underlying mechanisms of neurodegenerative diseases remain poorly understood. At present, symptom control is the primary treatment objective.^6^ It is estimated that neurodegenerative diseases will become the second most common cause of death.^7^

Alzheimer’s disease and dementias are in the top-ten ranking leading cause of death globally.^8^ Deposition of the insoluble and phosphorylated β-amyloid peptide (derived from amyloid precursor protein) in the brain parenchyma of Alzheimer’s disease patients affects functions/regeneration of various forms of neurons.^9^ The resulting widespread neuron damage affects synaptic communication leading to cognitive deficits, regional brain shrinkage, and brain atrophy;^10^ Huntington’s disease could appear in childhood or adolescence. Aberrant expansion of DNA segment containing CAG trinucleotide repeats in the huntingtin gene is a hallmark feature.^11^ Large CAG repeat is associated with early symptoms manifestation.^12^ Symptoms include poor coordination, chorea (involuntary dance-like movements), slow movement, seizures, and slurred speech; Parkinson’s disease affects motion control. Rigidity, tremor, and slow movement (bradykinesia) are frequently observed. Risk factors include genetic polymorphism, chronic inflammation, and metabolic disorders.^13^ Multiple sclerosis is a relapsing-remitting disease caused by an autoimmune attack in the central nervous system. Damage of myelin sheath in multiple areas by immune cells causes cognitive impairment, fatigue, muscle weakness, tremor, and vision problems.^14^

Brain disorders are frequently associated with mental/psychiatric illnesses.^15^ Mental illness is burdening the healthcare system with enormous unmet medical needs.^16,17^ Serious mental illness is closely linked to reduced life expectancy due to a higher risk of cardiovascular morbidity and mortality.^18^ Common mental illnesses include anxiety, depression, bipolar disorder, attention deficit hyperactivity disorder, and schizophrenia. Both children and adolescents are vulnerable to mental illnesses. Mental health condition is interlinked with physical health. The generation of suicide ideation/attempts and self-destructive thoughts are closely related to psychiatric diseases.^19^ Patients with degenerative diseases could also present emotional symptoms adding complexity to disease diagnosis and management. Recent studies reveal that hospitalized patients with COVID-19 and survivors display different levels of neuropsychiatric complications and the underlying mechanisms remain to be explored.^20^

GPCRs are one of the most intensively exploited targets for drug development. Approximately 35% of the FDA-listed drugs act through GPCRs.^21,22^ With our increasing understanding of the neuronal relay functions of GPCRs in the nervous system, many GPCRs are perceived as promising druggable targets for neurodegenerative and psychiatric diseases. This review summarizes the multifaceted role of GPCRs in chronic neurodegenerative conditions exemplified by Alzheimer’s disease, Huntington’s disease, Parkinson’s disease, and Multiple sclerosis. The emerging role of GPCRs on psychiatric illnesses, including Schizophrenia, Bipolar disorder, Depression, Attention deficit hyperactivity disorder, and Tourette’s disorder, are discussed. We also highlight the emerging opportunities for the previously unexplored GPCRs and provides examples of pharmaceutical development of GPCR-targeted therapeutics.

## G protein-coupled receptors signaling

Synaptic transmission can be classified into two types: fast and slow synaptic transmission.^23^ In fast synapses, GPCRs such as glutamate and GABA (γ-aminobutyric acid) receptors generate membrane depolarizing signals in less than 1/1000 s. In slow synapses, biogenic amines, peptides, and amino acid receptors generate signals in hundreds of milliseconds to minutes.^23^ GPCRs are structurally similar membrane proteins (Fig. 1). They elicit different intracellular signal pathways by interacting with heterotrimeric G proteins (α, β, and γ). GPCRs can be stabilized by an array of neurotransmitters and neurological modulators, including ions, hormones (peptide or non-peptide), vitamins, metabolites (ATP, fatty acids, etc.), natural products, and pharmacological ligands.^24^ A plethora of GPCR signaling events are involved in developing neuropsychiatric disorders. Understanding the downstream signaling events of disease-associated GPCR is essential for designing efficacious therapy.

Human GPCR can be classified into five distinct subtypes: rhodopsin (class A), secretin (class B1), adhesion (class B2), glutamate (class C), and frizzled (class F).^1^ To date, over 750 ligand-bound or apo-GPCR structures (including 96 CNS-related GPCRs) have been reported (Table 1). For details: https://gpcrdb.org. The transmembrane helical core exhibits high similarity. The helical core forms the orthosteric binding pocket for cognate ligands. GPCR can be divided into three different functional regions: (1) extracellular region including N-terminus, extracellular loops (ECLs), and extracellular ends of the transmembrane helices are involved in ligand recognition and selectivity;^25^ (2) intracellular region consisting of C-terminus, intracellular loops (ICLs) and intracellular ends of the transmembrane helices provide docking cavity for G proteins/ arrestins and interacts with different regulatory proteins such as GPCR kinases;^26^ (3) helical core in-between extracellular and intracellular region deliver and covert ligand signals via unique conformational change (Fig. 1b).^27,28^

**Table 1:** Reported GPCR structures

Class	Receptors	Total number	PDB ID (receptor alone)	PDB ID (G protein coupled receptor)
A	ADRB3	1		7DH5
	AGTR1	6	6OS0, 6OS1, 6OS2, 6DO1, 4ZUD, 4YAY	
	AGTR2	7	7JNI, 7C6A, 6JOD, 5XJM, 5UNH, 5UNG, 5UNM	
	HTR1A	3		7E2X, 7E2Y, 7E2Z
	HTR1B	5	7C61, 5V54, 4IAR, 4IAQ	6G79
	HTR1D	1		7E32
	HTR1E	1		7E33
	HTR1F	1		7EXD
	HTR2A	13	7WC4, 7WC5, 7WC6, 7WC7, 7WC8, 7WC9, 7VOD, 7VOE, 6WGT, 6WH4, 6A94, 6A93	6WHA
	HTR2B	8	6DRY, 6DS0, 6DRZ, 6DRX, 5TUD, 5TVN, 4NC3, 4IB4	
	HTR2C	2	6BQG, 6BQH	
	ACM1	6	6ZFZ, 6ZG9, 6ZG4, 6WJC, 5CXV	6OIJ
	ACM2	10	5ZKB, 5ZKC, 5ZK3, 5ZK8, 5YC8, 4MQT, 4MQS, 3UON	6U1N, 6OIK
	ACM3	5	5ZHP, 4U14, 4U15, 4U16, 4DAJ	
	ACM4	2	6KP6, 5DSG	
	ACM5	1	6OL9	
	APJ	2	6KNM, 5VBL	
	BKRB1	1		7EIB
	BKRB2	1		7F2O
	C5AR1	3	6C1Q, 6C1R, 5O9H	
	CCKAR	8	7F8X, 7F8U, 7F8Y	7EZM, 7EZH, 7EZK, 7MBX, 7MBY
	CCR1	3		7VLA, 7VL8, 7VL9
	CCR2	3		6GPS, 6GPX, 5T1A
	CCR5	11	7F1T, 6MET, 6MEO, 6AKY, 6AKX, 5UIW, 4MBS	7F1Q, 7F1R, 7F1S, 7O7F
	CCR6	1		6WWZ
	CCR7	1	6QZH	
	CCR9	1	5LWE	
	CNR1	8	7V3Z, 6KQI, 5XRA, 5XR8, 5U09, 5TGZ	6KPG, 6N4B
	CNR2	4	6KPC, 5ZTY	6KPF, 6PT0
	CXCR2	3	6LFL	6LFM, 6LFO
	CXC-R4	6	4RWS, 3ODU, 3OE0, 3OE6, 3OE8, 3OE9	
	DRD1	11		7JOZ, 7CKW, 7CKX, 7CKY, 7CKZ, 7CRH, 7LJC, 7LJD, 7JV5, 7JVP, 7JVQ
	DRD2	5	7DFP, 6LUQ, 6CM4	7JVR, 6VMS
	DRD3	3	3PBL	7CMV, 7CMU
	DRD4	3	6IQL, 5WIV, 5WIU	
	EDNRB	8	6LRY, 6K1Q, 6IGL, 6IGK, 5XPR, 5X93, 5GLH, 5GLI	
	FFAR1	4	5KW2, 5TZY, 5TZR, 4PHU	
	FPR1	2		7WVU, 7T6T
	FPR2	9	6LW5	7WVV, 7WVX, 7WVW, 7WVY, 7T6V, 7T6S, 7T6U, 6OMM
	GALR1	1		7WQ3
	GALR2	1		7WQ4
	CCKBR	2		7F8V, 7F8W
	GHSR	7	7F83, 6KO5	7W2Z, 7NA7, 7NA8, 7F9Y, 7F9Z
	GNRHR	1	7BR3	
	GPBAR1	3		7CFM,7CFN, 7BW0
	HRH1	2	3RZE	7DFL
	LPAR1	6	4Z34, 4Z35, 4Z36	7TD0, 7TD1, 7TD2
	LSHR	4	7FIJ	7FIG, 7FII, 7FIH
	LT4R1	2	7K15	7VKT
	MC4R	8	6W25	7PIV, 7PIU, 7F53, 7F54, 7F55, 7F58, 7AUE
	MSHR	4		7F4D, 7F4H, 7F4I, 7F4I
	MTNR1A	8	6PS8, 6ME2, 6ME3, 6ME4, 6ME5	7VGY, 7VGZ, 7DB6
	MTNR1B	5	6ME6, 6ME7, 6ME8, 6ME9	7VH0
	NK1R	11	6J20, 6J21, 6HLP, 6HLL, 6HLO, 6E59	7P00, 7P02, 7RMI, 7RMG, 7RMH
	NPY1R	3	5ZBH, 5ZBQ	7VGX
	NPY2R	1	7DDZ	
	NTSR1	24	6YVR, 6Z4Q, 6Z4S, 6Z4V, 6Z66, 6Z8N, etc	7L0P, 7L0Q, 7L0R, 7L0S, 6UP7, 6PWC, 6OSA, 6OS9
	OPRD	6	6PT2, 6PT3, 4RWD, 4RWA, 4N6H, 4EJ4	
	OPRK	3	6VI4, 6B73, 4DJH	
	OPRM	7	5C1M, 4DKL	7U2L, 7SBF, 7SCG, 6DDF, 6DDE
	OPRL1	3	5DHG, 5DHH, 4EA3	
	HCRTR1	14	6V9S, 6TOT, 6TOS, 6TOD, 6TQ4, 6TP4, etc	
	HCRTR2	8	6TPG, 6TPJ, 6TPN, 5WS3, 5WQC, 4S0V	7L1U, 7L1V
	OXYR	2	6TPK	7RYC
	P2RY1	2	4XNV, 4XNW	
	P2Y12	3	4PXZ, 4PY0, 4NTJ	
	PAR1	1	3VW7	
	PAR2	3	5NDZ, 5NJ6, 5NDD	
	PTGDR2	3	7M8W, 6D26, 6D27	
	PTGER2	3		7CX2, 7CX3, 7CX4
	PTGER3	2	6AK3, 6M9T	
	PTGER4	3	5YHL, 5YWY	7D7M
	PTAFR	2	5ZKQ, 5ZKP	
	lpar6a	1	5XSZ	
	BLT1	1	5X33	
	S1PR1	11	3V2W, 3V2Y	7TD4, 7TD3, 7EO4, 7EO2, 7EVY, 7WF7, 7EW0, 7EW7, 7EVZ
	S1PR2	1		7T6B
	S1PR3	4	7C4S	7EW2, 7EW3, 7EW4
	S1PR5	1		7EW1
	SSR2	2		7T10, 7T11
	SUCR1	3	6Z10, 6RNK, 6IBB	
	TBXA2R	2	6IIU, 6IIV	
	V2R	2		7DW9, 7BB6
	GPR52	4	6LI0, 6LI1, 6LI2	6LI3
	GPR88	2		7EJX, 7WZ4
	GPR139	4		7VUH, 7VUJ, 7VUI, 7VUY
	GPR183	2	7TUY	7TUZ
	MRGX2	14	7VV6, 7VV4, 7VV0	7VDM, 7VDH, 7VUZ, 7VDL, 7VV5, 7VUY, 7VV3, 7S8M, 7S8O, 7S8L, 7S8N
	MRGX4	1		7S8P
B1	CALCR	12		5UZ7, 6NIY, 7TYL, 7TYI, 7TYN, 7TYO, 7TYF, 7TYW, 7TYH, 7TYX, 7TYY, 7TZF
	CALRL	6	7KNU, 7KNT	6E3Y, 6UVA, 6UUN, 6UUS
	CRFR1	4	4K5Y, 4Z9G	6PB0, 6P9X
	CRFR2	1		6PB1
	GHRHR	2		7CZ5, 7V9M
	GIPR	6		7FIY, 7VAB, 7FIN, 7DTY, 7RBT, 7RA3
	GLP1R	34	5NX2, 5VEW, 5VEX, 6KJV, 6KK1, 6KK7, 6LN2	7FIM, 7VBI, 7LLL, 7LLY, 7S1M, 7S3I, 7RTB, 7DUR, 7EVM, 7KI0, 7KI1, 7DUQ, 7E14, 7LCJ, 7LCK, 7LCI, 6XOX, 6X1A, 6X18, 6X19, 7C2E, 6VCB, 6ORV, 6B3J, 7RGP, 7RG9, 7VBH
	GLP2R	1		7D68
	GCGR	10	4L6R, 5EE7, 5XEZ, 5XF1, 5YQZ	6LMK, 6LML, 6WHC, 6WPW, 7V35
	SCTR	3		6WZG, 6WI9, 7D3S
B2	ADGRG1	1		7SF8
	ADGRL3	1		7SF7
C	GABBR1	1	6W2Y	
	GABBR2	12	7C7S, 7C7Q, 6UO8, 6VJM, 6UOA, 6UO9, 6W2X, 6WIV, 7CUM, 7CA5, 7CA3	7EB2
	GRM1	3	4OR2, 7DGE, 7DGD,	
	GRM2	9	7MTR, 7MTQ, 7EPE, 7EPD, 7EPB, 7EPF, 7EPA	7MTS, 7E9G
	GRM3	3	7WI6, 7WI8, 7WIH	
	GRM4	1		7E9H
	GRM5	11	4OO9, 5CGC, 5CGD, 6FFH, 6FFI, 6N4X, 6N4Y, 6N50, 6N51, 6N52, 7FD8, 7P2L, 7FD9	
	GRM7	1	7EPC	
	GP158	5	7EWL, 7SHF, 7SHE, 7EWR, 7EWP	
	CASR	16	7SIL, 7SIM, 7SIN, 7E6U, 7E6T, 7M3E, 7M3J, 7M3G, 7M3F, 7DD5, 7DD6, 7DD7, 7DTU,7DTW, 7DTV, 7DTT	
F	FZD4	1	6BD4	
	FZD5	1	6WW2	
	FZD7	1		7EVW

*ADRB3* beta-3 adrenergic receptor, *AGTR1* type 1 angiotensin II receptor, *AGTR2* type 2 angiotensin II receptor, *HTR1A* 5-hydroxytryptamine receptor 1A, *HTR1B* 5-hydroxytryptamine receptor 1B, *HTR1D* 5-hydroxytryptamine receptor 1D, *HTR1E* 5-hydroxytryptamine receptor 1E, *HTR1F* 5-hydroxytryptamine receptor 1F, *HTR2A* 5-hydroxytryptamine receptor 2A, *HTR2B* 5-hydroxytryptamine receptor 2B, *HTR2C* 5-hydroxytryptamine receptor 2C, *ACM1* muscarinic acetylcholine receptor M1, *ACM2* muscarinic acetylcholine receptor M2, *ACM3* muscarinic acetylcholine receptor M3, *ACM4* muscarinic acetylcholine receptor M4, *ACM5* muscarinic acetylcholine receptor M5, *APJ* apelin receptor, *BKRB1* B1 bradykinin receptor, *BKRB2* B2 bradykinin receptor, *C5AR1* C5a anaphylatoxin chemotactic receptor 1, *CCKAR* cholecystokinin receptor type A, *CCR1* cinnamoyl-CoA reductase 1, *CCR2* C-C chemokine receptor type 2, *CCR5* C-C chemokine receptor type 5, *CCR6* C-C chemokine receptor type 6, *CCR7* C-C chemokine receptor type 7, *CCR9* C-C chemokine receptor type 9, *CNR1* cannabinoid receptor 1, *CNR2* cannabinoid receptor 2, *CXCR2* C-X-C chemokine receptor type 2, *CXC-R4* C-X-C chemokine receptor type 4, *DRD1* D(1A) dopamine receptor, *DRD2* D(2) dopamine receptor, *DRD3* D(3) dopamine receptor, *DRD4* D(4) dopamine receptor, *EDNRB* endothelin receptor type B, *FFAR1* free fatty acid receptor 1, *FPR1* fMet-Leu-Phe receptor, *FPR2* N-formyl peptide receptor 2, *GALR1* galanin receptor type 1, *GALR2* galanin receptor type 2, *CCKBR* gastrin/cholecystokinin type B receptor, *GHSR* growth hormone secretagogue receptor type 1, *GNRHR* gonadotropin-releasing hormone receptor, *GPBAR1* G-protein coupled bile acid receptor 1, *HRH1* histamine H1 receptor, *LPAR1* lysophosphatidic acid receptor 1, *LSHR* lutropin-choriogonadotropic hormone receptor, *LT4R1* leukotriene B4 receptor 1, *MC4R* melanocortin receptor 4, *MSHR* melanocyte-stimulating hormone receptor, *MTNR1A* melatonin receptor type 1A, *MTNR1B* melatonin receptor type 1B, *NK1R* substance-P receptor, *NPY1R* neuropeptide Y receptor type 1, *NPY2R* neuropeptide Y receptor type 2, *NTSR1* neurotensin receptor type 1, *OPRD* delta-type opioid receptor, *OPRK* kappa-type opioid receptor, *OPRM* mu-type opioid receptor, *OPRL1* nociceptin receptor, *HCRTR1* orexin/hypocretin receptor type 1, *HCRTR2* orexin receptor type 2, *OXYR* oxytocin receptor, *P2RY1* P2Y purinoceptor 1, *P2Y12* P2Y purinoceptor 12, *PAR1* proteinase-activated receptor 1, *PAR2* proteinase-activated receptor 2, *PTGDR2* prostaglandin D2 receptor 2, *PTGER2* prostaglandin E2 receptor EP2 subtype, *PTGER3* prostaglandin E2 receptor EP3 subtype, *PTGER4* prostaglandin E2 receptor EP4 subtype, *PTAFR* platelet-activating factor receptor, *lpar6a* lysophosphatidic acid receptor 6a, *BLT1* leukotriene B4 receptor 1, *S1PR1* sphingosine 1-phosphate receptor 1, *S1PR2* sphingosine 1-phosphate receptor 2, *S1PR3* sphingosine 1-phosphate receptor 3, *S1PR5* sphingosine 1-phosphate receptor 5, *SSR2* somatostatin receptor type 2, *SUCR1* succinate receptor 1, *TBXA2R* thromboxane A2 receptor, *V2R* vasopressin V2 receptor, *GPR52* G-protein coupled receptor 52, *GPR88* probable G-protein coupled receptor 88, *GPR139* probable G-protein coupled receptor 139, *GPR183* G-protein coupled receptor 183, *MRGX2* Mas-related G-protein coupled receptor member X2, *MRGX4* Mas-related G-protein coupled receptor member X4, *CALCR* calcitonin receptor, *CALRL* calcitonin gene-related peptide type 1 receptor, *CRFR1* corticotropin-releasing factor receptor 1, *CRFR2* corticotropin-releasing factor receptor 2, *GHRHR* growth hormone-releasing hormone receptor, *GIPR* gastric inhibitory polypeptide receptor, *GLP1R* glucagon-like peptide-1 receptor, *GLP2R* glucagon-like peptide 2 receptor, *GCGR* glucagon receptor, *SCTR* secretin receptor, *ADGRG1* adhesion G-protein coupled receptor G1, *ADGRL3* adhesion G protein-coupled receptor L3, *GABR1* gamma-aminobutyric acid type B receptor subunit 1, *GABBR2* gamma-aminobutyric acid type B receptor subunit 2, *GRM1* metabotropic glutamate receptor 1, *GRM2* metabotropic glutamate receptor 2, *GRM3* metabotropic glutamate receptor 3, *GRM4* metabotropic glutamate receptor 4, *GRM5* metabotropic glutamate receptor 5, *GRM7* metabotropic glutamate receptor 7, *GP158* probable G-protein coupled receptor 158, *CASR* extracellular calcium-sensing receptor, *FZD4* Frizzled-4, *FZD5* Frizzled-5, *FZD7* Frizzled-7

**Fig. 2:**
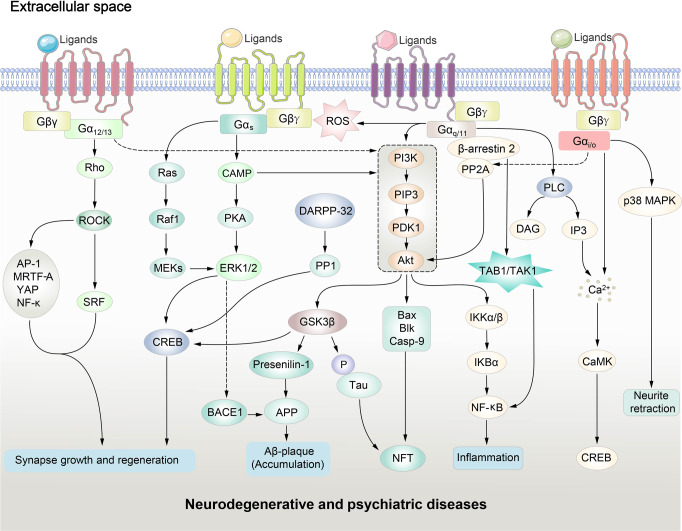
GPCR-regulated downstream signaling pathways in neurodegenerative and psychiatric disorders. CAMK calmodulin-dependent protein kinase, BACE1 β-site APP cleaving protein 1, TAK1 transforming growth factor-β-activated kinase, TAB1 TAK1 binding protein, PP2A protein-phosphatase 2A, PLC phospholipase C, PDK1 phosphoinositide-dependent kinase 1, diacylglycerol (DAG), IP3 inositol triphosphate, Akt protein kinase B, APP amyloid protein precursor, Bax B-cell lymphoma-2-associated X, Blk B lymphoid tyrosine kinase, cAMP cyclic adenosine monophosphate, Casp9 caspase 9, CREB cAMP response element binding protein, ERK1/2 extracellular signal-regulated-kinase, GSK3β glycogen synthase kinase 3β, Gβγ free heterotrimeric G protein beta/gamma subunits, IKBα inhibitory subunit of nuclear factor kappa-B alpha, IKKα/β inhibitor of kappa-B kinase, MEK mitogen-activated protein, NFT neurofibrillary tangles, NF-κB nuclear factor kappa-B, PI3K phosphoinositide 3-kinase, PKA protein kinase A, Raf1 Raf-1 proto-oncogene, serine/threonine kinase, Ras Ras Sarcoma oncoproteins, Rho Ras homologous proteins, ROCK Rho-associated coiled-coil containing kinases, SRF serum response factor

Activated receptors generate second messengers via the G protein. In heterotrimeric form, the G protein is inactive. After binding to the intracellular cavity formed by GPCR, the GDP-binding pocket on the Gα subunit of heterotrimeric G proteins is opened, facilitating subsequent exchange for GTP.^29^ GTP is physiologically more abundant as compared to GDP.^30^ The nucleotide exchange is a rate-limiting step in the G protein activation process.^29^ GTP binding prevents Gα protein from forming heteromer with Gβγ subunit.^31^ The free Gα and Gβγ subunits modulate different downstream effector pathways. By hydrolyzing GTP to GDP, the active GTP-bound Gα subunit returns to an inactive state and forms a complex with the Gβγ subunit again. G proteins are classified based on their Gα subunit. There are four different Gα protein families: Gαi/o, Gαs, Gαq/11, and Gα12/13. Each family regulates a specific set of downstream responses. Individual GPCR could mediate different functions in different cellular contexts via preferential G protein coupling (Figs. 1 and 2).

### Gα proteins: Gαs and Gαi/o

Gαs (stimulatory regulator of adenylyl cyclase G protein activates adenylyl cyclase) promotes the generation of 3’-5’-cyclic adenosine monophosphate (cAMP) from ATP by adenylate cyclase. cAMP is essential for protein kinase A (PKA)-mediated signal transduction;^32^ In contrast, Gαi/o suppresses adenylyl cyclase activity, which prevents cAMP accumulation and reduces PKA activity. cAMP is a crucial regulator of the phosphoinositide 3-kinase/AKT murine thymoma viral oncogene homolog (PI3K/AKT) signaling pathway. It has been shown that PI3K/AKT is associated with the inflammatory response in multiple neurodegenerative diseases.^33–35^ cAMP is also linked to calcium dynamics in neuronal cells and neurodegenerative diseases. Details can be found in the comprehensive review by Sobolczyk and Boczek.^36^

### Gα protein: Gαq/11

Gαq activates phospholipase C (PLC), which hydrolyzes phosphatidylinositol 4,5-biphosphate into diacylglycerol (DAG) and inositol 1,4,5-trisphosphate (IP3). DAG activates protein kinase C, which phosphorylates various downstream signaling proteins. IP3 stimulates calcium efflux from the endoplasmic reticulum through specific IP3 receptors. Calcium signaling is essential for the release of neurotransmitters.^37,38^ For instance, dysregulation of the dopamine D1 receptor-mediated PLC/IP3/Ca^2+^ pathway in the anterior cortex of the brain is associated with mental illness in rats.^39,40^ PLC/IP3/Ca^2+^ pathway regulates the electrical response of the neuron.^41^ Impaired Ca^2+^ homeostasis by Aβ exposure is one of the underlying causes of amyloid toxicity in Alzheimer’s disease.^42^ In psychiatric disorders, Ca^2+^ signaling regulates neuronal connectivity, synaptic plasticity, and glial functions.^43^

### Gα protein: Gα12/13

Gα12/13 binding can stimulate Rho family GTPases.^44^ Rho GTPases activate the cytosolic Rho protein by promoting GDP/GTP exchange.^45^ Activated Rho is released from inhibitory protein, migrates to the plasma membrane, and modulates multiple downstream effectors.^46^ One of which is ROCK1/2 (Rho kinase). The Rho-ROCK pathway is essential in neurodegenerative diseases, including Alzheimer’s disease, Parkinson’s disease, amyotrophic lateral sclerosis, and Huntington’s disease.^47^ ROCK activity is closely associated with neuronal cell loss, impaired synaptic functions, and cytoskeleton modulation in central nervous system disorders.^47^ Rho/ROCK signaling modulates the activity of transcription regulators such as AP-1, MRTF-A, YAP, NF-κB, and serum response factor.^47,48^ Rho family GTPases are essential for axon guidance, cell polarity, and synapse formation.^49^ It has been shown that Rho GTPase regulates neuronal cell survival by inhibiting AKT signaling.^50^

### GPCR kinases (GRKs)

Activated GPCR is subjected to desensitization to protect the cell from sustained stimulation.^51^ After peak response, ligand-bound receptor activity will return to basal level.^52^ Receptor phosphorylation by a family of GPCR kinases (GRKs), including GRK1/7, GRK2/3, and GRK4/5/6, is an essential first step to switch off sustained signaling.^53,54^ GRKs are second messenger-independent kinases (e.g., in contrast to PKA, which is dependent on cAMP levels). Serine/threonine residues on the GPCR carboxyl-terminal tail are common phosphorylation sites targeted by GRKs.^55^ GRKs translocate from cytoplasm to plasma membrane and initiate receptor phosphorylation by binding to Gβγ.^56,57^ GRK could also interfere with G protein binding through direct interaction.^58^ GRK level is affected by inflammatory responses in neonatal and adult neurons.^59^ GRK dysfunction is associated with cognitive impairment and tau hyperphosphorylation in Alzheimer-like pathology.^60^ Colocalization of GRK with amyloid plaques is observed in brain tissues of Alzheimer’s disease patients.^61^ Patients of Parkinson’s disease with dementia have increased GRK3/5 transcripts.^62^ GRK might promote the formation of pathological Lewy bodies in sporadic Parkinson’s disease, but the mechanism is yet to be defined.^63^ In psychiatric disorders, upregulating brain GRKs are observed in schizophrenia and major depression.^64,65^

### Arrestins in GPCR desensitization

**Fig. 3:**
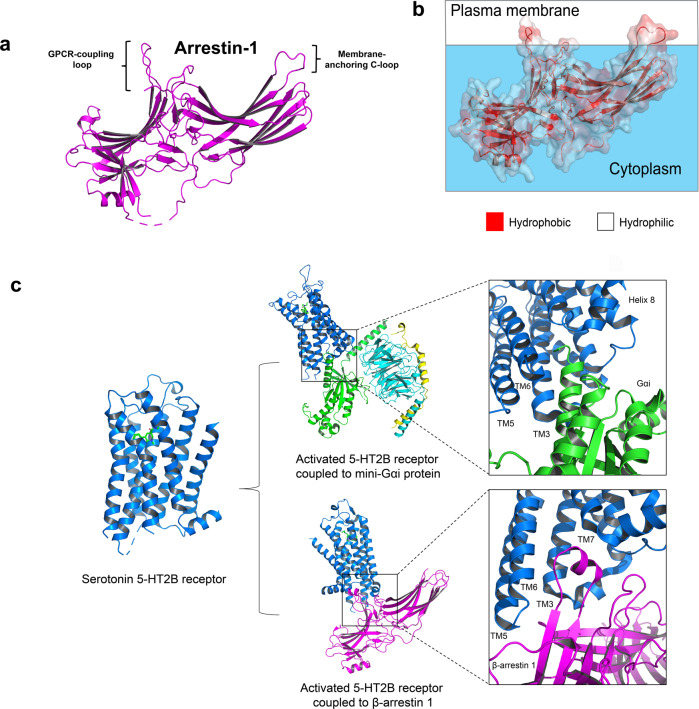
GPCR-G protein/arrestin complexes. **a** Crystal structure of arrestin 1 (PDB 1CF1) showing the membrane-anchoring c-loop. **b** Solvent-accessible surface. Hydrophobic surface (red); Hydrophilic surface (white). **c** Biased signaling of serotonin 5-HT2B receptors. Activated 5-HT2B receptor (PDB 7SRQ) is preferentially coupled to Gαs protein (PDB 7SRR). The receptor could also couple to β-arrestin 1 (PDB 7SRS). Gαs and β-arrestin 1 engaged on the same cavity formed by the cytoplasmic receptor interface

Active GPCR is ready for the arrestins (signal terminators) binding after GRK phosphorylation. Arrestins can be classified into visual arrestins (arrestin 1 and arrestin 4) and non-visual arrestins (β-arrestin 1/2 or arrestin 2/3). Visual arrestins express exclusively in retina photoreceptors. They regulate light-activated rhodopsin signaling.^66,67^ β-arrestin 1/2 are ubiquitously expressed cytoplasmic proteins (Fig. 3a, b).^52^ β-arrestins and G proteins compete for the receptors. They bind to the same inter-helical cavity on the intracellular region (Fig. 3c).^68^ β-arrestin reduce G protein singling by hindering interaction between receptor and heterotrimeric G proteins. Further, β-arrestins facilitate receptor recycling by promoting internalization and cellular trafficking.^69,70^ The C-edge of arrestin protein with proximity to the membrane surface functions membrane anchor to stabilize the arrestin-active receptor complex (Fig. 3a).^71^ Recent studies illustrate the association of β-arrestin in multiple physiological functions and neuropsychiatric disorders.^72,73^ Phosphorylation of PI3K/AKT is remarkably reduced in the β-arrestin 2-deficient adult neural stem cells, indicating the crucial role of β-arrestin 2-PI3K/Akt pathway in adult hippocampal neurogenesis.^74,75^

### Biased signaling of GPCRs

G protein-biased signaling is regarded as the canonical signaling pathway employed by GPCRs.

β-arrestin can modulate GPCR signal transduction in G protein-independent mechanism. β-arrestin can use the receptor as a structural component to generate an intracellular signaling complex consisting of agonist-occupied receptor and nonreceptor tyrosine kinases (c-Src).^76^ β-arrestin can maintain ERK signaling by acting as a scaffold for ERK mitogen-activated protein kinase.^77^ Other downstream effectors of β-arrestins include phosphatases and transcription factors.^78^ β-arrestins can act as a scaffold protein for specific downstream effectors.^79,80^ In the mouse model, β-arrestin 2 exerts anti-inflammatory functions by inhibiting nuclear factor kappa-B.^81^ Maintaining the arrestin-dependent signaling of M1 muscarinic acetylcholine receptor can prevent the insoluble misfolded proteins accumulation in Alzheimer’s disease model which thereby slowing down neurodegenerative disease progression.^82^

β-arrestin is important for astrocyte-mediated pro-inflammatory cytokine production.^83^ In mouse Parkinson’s disease models, β-arrestin 2-biased ligands suppress glia-derived inflammation and prevent neuron loss.^84^ IL-1β produced by the inflammation site is suppressed by β-arrestin 2.^84^ As compared to agonists which facilitate G protein and β-arrestin signaling at the same time, a β-arrestin-biased agonist for δ-opioid receptor can effectively control anxiety-like behaviour by activating ERK1/2 in the limbic structures of the brain.^85^ Hence, identifying therapeutic modulators that could preferentially stabilize GPCR structure for G proteins or β-arrestins is important for developing effective treatments for neurodegenerative and psychiatric diseases.

### Examples of GPCR-regulated modulators in disease development

#### β-site APP cleaving protein 1 (BACE1)

The proteolytic activity of BACE1 promotes the generation of β-amyloid (Aβ) peptides from amyloid precursor protein in Alzheimer’s disease.^86^ BACE1 expression can be activated by muscarinic acetylcholine receptor M1/M3 via PKC and MAP kinase signaling cascades.^87^ BACE1 activity is modulated by other GPCRs, such as the A2A and delta-opioid receptors.^33^ It has been shown that selective activation of the M2 receptor will suppress BACE1 expression via PKA-mediated signaling events.^33^

#### cAMP-response element binding protein (CREB)

GWAS analysis indicates that genes involved in the cAMP/PKA/CREB pathway are genetically associated with schizophrenia and bipolar disorder.^88^ CREB is a transcription factor activated by phosphorylation after GPCR activation. The binding of CREB to a specific cAMP response element (CRE) in the transcription regulatory region enhances particular gene transcription. For instance, neurotransmitter-activated dopamine D1 receptor on dopaminergic neurons can elicit transcription brain derived growth factor (BDNF) and other neurotrophins.^89^ In patients with bipolar disorder and schizophrenia, CREB expression is remarkably reduced in the dorsolateral prefrontal cortex and cingulate gyrus.^90^ While CREB protects neuronal cells in neurogenerative diseases, constitutively active CREB can reduce hippocampal neuron numbers and trigger sporadic epileptic seizures.^91,92^ It has been shown that the CREB modulator could enhance synaptic plasticity, which is beneficial for schizophrenia treatment.^89^

#### DARPP-32 and PP1

DARPP-32 (dopamine- and cyclic-AMP-regulated phosphoprotein of molecular weight 32,000) regulates neuronal excitability levels by prolonged depolarizations and voltage oscillations.^93^ DARPP-32 is the downstream target of Gi-coupling receptors such as the D2 dopamine receptor. DARPP-32 functions as a protein phosphatase-1 (PP1) inhibitor, a eukaryotic serine/threonine protein, upon phosphorylation at Thr-34 by PKA. PP1 is a phosphatase with multiple physiological functions. PP1 controls clock component PER2 accumulation in neurons, influencing circadian rhythm by light-mediated clock resetting.^94^ PP1 is an inducer of long-term synaptic depression in the hippocampus.^95^ Dysregulation of glutamate and dopamine signaling is common in neurodegenerative and neuropsychiatric disorders. Quantitative modeling results suggested that DARPP-32 could integrate dopamine and glutamate signals in striatal neurons.^96^ PP1 signaling reduces GABA(A) receptors in neostriatal medium spiny neurons depending on PKA and DARPP.^97^

## GPCRs in neuropsychiatric diseases

### Class A GPCR (rhodopsin)

#### Structural insights

**Fig. 4:**
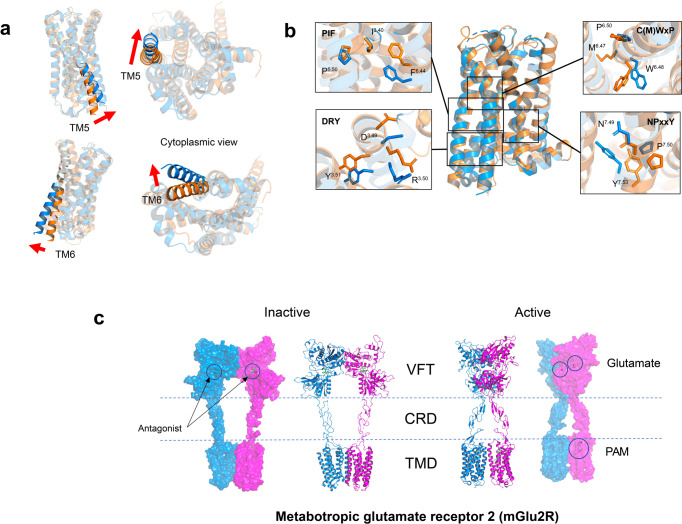
Class A GPCR activation. **a** Prominent outward bending of TM5 and TM6 opens the cytoplasmic pocket of inactive serotonin 5-HT2A receptor (orange, PDB 6A93) for the binding of G protein. Active 5-HT2A receptor (marine blue, PDB 6WHA). **b** Microswitches involved in 5-HT2A receptor activation. **c** Structural features of class C GPCRs. Inactive (PDB 7MTQ) and active metabotropic glutamate receptor 2 mGlu2R (PDB 7MTR). VFT extracellular venus flytrap domain, CRD cysteine-rich domain, TMD transmembrane domain, PAM positive allosteric modulator

Class A GPCR is the most heavily investigated GPCR family for drug development. Ligand binding to the unique pocket stabilizes GPCR in a particular conformation.^98^ Comparative analysis reveals that the outward bending/rotation of intracellular TM6 is a universal structure feature of receptor activation throughout the GPCR superfamily (Fig. 4a).^98^ Hydrophobic packing interactions between the transmembrane helices help to maintain the active conformation of TM6.^99^ Apart from TM6, rearrangements of other transmembrane helices, including TM3/5/7, open the intracellular milieu to facilitate recruitment of G protein.^100^ Class A GPCR has a consensus binding interface for G protein coupling.^101^ The receptors employ unique structure motifs as microswitches to transmit external stimuli (Fig. 4b). D^3.49^R^3.50^Y^3.51^ motif (Ballesteros–Weinstein number) at the intracellular region of TM3 forms the classic “ionic lock” with E^6.30^ on TM6 to constrain the receptor in the ground state.^102,103^ Disruption of the ionic lock is an activation feature of class A GPCRs.^104^ Side chains of Y^7.53^ (NPxxY motif) on TM7 and W^6.48^ (CWxP motif) on TM6 are subjected to orientation rearrangement during receptor activation.^105,106^ The P^5.50^I^3.40^F^6.44^ motif, formed by a group of hydrophobic residues on TM3/5/6, is also a crucial switch for receptor activation.^107^ Polar interactions and aromatic stacking interactions between the conserved aromatic residues are frequently observed in the ligand binding region of activated class A GPCRs.^27^

#### Acetylcholine receptors (muscarinic)

Acetylcholine is a neurotransmitter employed by cholinergic neurons in the brain and spinal cord.^108^ Muscarinic acetylcholine receptors in the central and peripheral nervous systems have five distinct subtypes. M1, M3, and M5 receptors are excitatory M1-like receptors.^109^ In contrast, M2-like receptors (M2 and M4 receptors) inhibit adenylyl cyclase activity. All the subtypes are detected in the brain. M2 and M3 receptors are also found in peripheral tissues.^110^

Reduced acetylcholine signaling due to the loss of cholinergic neurons is common in Alzheimer’s disease.^111^ Amyloid-β proteins could interrupt the interaction between the M1 receptor and G protein.^112^ M1 receptor-knockout mice show Alzheimer’s disease-like pathology with age-dependent cognitive decline.^113^ M1 receptor function is impaired by the binding of tau protein, a microtubule-associated protein in the extracellular matrix, which is toxic in secreted form;^114,115^ Autoantibodies to recombinant human M1 receptors are detected in patients with schizophrenic disorders, mood disorders, and other psychiatric disorders.^116,117^

The M1 receptor is a promising target for schizophrenia treatment. Allosteric modulation of M1 receptor activity could improve cognitive performance with antipsychotic activity.^118^ However, substantial loss of cortical M1 receptor might affect the efficacy of positive allosteric modulator.^119^

M2 receptor reduction is noted in the frontal cortex of Alzheimer’s disease patients.^120^ Suppressing M2 receptor expression with siRNA alters the expression of β-site APP cleaving protein. This transmembrane aspartic endopeptidase is involved in beta-amyloid formation.^121^

M2 receptor is suspected to be related to the major depressive disorder and bipolar disorder development.^122^ M2-encoding gene is genetically associated with the cholinergic dysfunction seen in mood disorders.^122^

M3 receptor level is remarkably reduced in the post-mortem frontal cortex tissues of patients with bipolar disorder.^123^ However, conflicting results are observed in another study cohort.^124^ Genetic variants of the M3 receptor-encoding gene are associated with abnormal neural connectivity in schizophrenia and cannabis-induced hallucinations.^125,126^

Acetylcholine elevation is observed in Parkinson’s disease.^127–129^ Targeting the M4 receptor with various antagonists showed promising treatment results for Parkinson’s disease.^130,131^ M4 receptor is abundantly expressed in striatal neurons, which regulates the balance between acetylcholine and dopamine responses.^132^ M4 receptor promotes the development of the dopamine hypersensitivity phenotype of schizophrenia.^133^ It has been shown that the M5 receptor can potentiate drug addiction by reinforcing rewarded behavior.^134^

#### Adenosine receptor

Adenosine (A1A, A2A, A2B, A3A) receptors are synaptic modulators that transmit inhibitory signals from adenosine to excitatory synapses.^135^ Adenosine is also known as a “retaliatory metabolite” as it is produced exponentially from tissue under stress.^136^ Astrocytes release adenosine to modulate synaptic transmission during hypoxia.^137^ A1A and A2A receptors exhibit widespread expression in the brain.^138^ A1A and A3A receptors are Gi-coupling receptors. In contrast, A2A and A2B receptors prefer Gs for downstream signaling.

Although dopamine-replacement therapy is the mainstay treatment for Parkinson’s disease, it remains challenging to manage dyskinesia during replacement treatments.^139^ Animal study reveals that activating the A2A receptor will reduce the agonistic effects of dopaminergic D2 receptor-targeting drugs.^140^ As the A2A receptor is colocalized with D2 dopaminergic receptors, it is suggested that interactions between A2A and D2 receptors might be involved in the pathophysiology of Parkinson’s disease.^141^

Epidemiological data support that caffeine (a naturally occurring methylxanthine) consumption might reduce the risk of depression or depressive symptoms.^142,143^ The psychoactive function of caffeine is mediated via the non-selective antagonistic action on A1/A2A receptors.^144^ How A1/A2A receptors regulate depression-like behaviour remains unclear.^145^ It should be noted that caffeine at high doses might function other than adenosine receptor antagonists causing insomnia and anxiety.^146,147^

Activated A2A receptor suppresses nitric oxide (NO) production by inhibiting NO synthetase.^12^ NO signaling is associated with various neurodegenerative diseases, including Parkinson’s disease, amyotrophic lateral sclerosis, multiple sclerosis, amyotrophic lateral sclerosis, and Alzheimer’s disease.^148^ NO is a mediator of neuroinflammation, which triggers the microglial to release pro-inflammatory factors.^149^ NO induces protein S-nitrosylation (covalent addition of a NO group to a cysteine thiol/sulfhydryl), imposing endoplasmic reticulum stress in neurons.^150,151^ As A2A receptor activation affects synaptic plasticity and introduces memory deficits, antagonizing the A2A receptor might be helpful to control age-related cognitive impairments in Alzheimer’s disease.^152^

#### Adrenergic receptor

Brain adrenergic receptors on neurons and glia are activated by the monoamine neurotransmitter norepinephrine (produced primarily in the locus coeruleus of the brain stem) and epinephrine.^153^ Norepinephrine is produced from dopamine and converted into epinephrine. Norepinephrine and epinephrine released at synaptic junctions in the autonomic nervous system control classical fight-or-flight response.^154^ Norepinephrine controls response to environmental changes by regulating neuronal excitability.^155^ Epinephrine and norepinephrine also affect intelligence.^156^ Human has 2 adrenergic receptor subtypes: α-adrenergic (α1, α2A, α2B, α2C) receptors and β-adrenergic (β1, β2, β3) receptors. All the subtypes can be detected in the brain tissues.

Adrenergic receptor protects the central nervous system from uncontrolled inflammatory responses.^157^ In the neonatal Lewis rats model, norepinephrine protects neuronal damage from inflammation.^158–161^ Blocking β-adrenergic receptors signaling with beta-blockers (β-adrenergic antagonists) could exacerbate neuroinflammation in a mouse model of Alzheimer’s disease.^162^

Patients of Alzheimer’s disease and Parkinson’s disease show profound cell loss in locus coeruleus.^163^ Amyloid Aβ affects norepinephrine production and alters adrenergic receptor signaling in Alzheimer’s disease;^164^ Low norepinephrine level is linked to mood disorders such as anxiety, depression, and attention deficit hyperactivity disorder.^156^ α2-adrenergic receptors are established targets for antidepressant therapy.^165^ Depressed suicide victims showed high α2A-adrenergic receptor in the prefrontal cortex.^166^ Presynaptic α2-adrenergic receptor is an auto-receptor with the highest affinity to norepinephrine. Activated α2-adrenergic receptor inhibits norepinephrine synthesis and release.^167^ Thus, antagonizing presynaptic α2-adrenergic receptors could benefit depression treatment by enhancing norepinephrine release.^168^

#### Cannabinoid receptor

Cannabinoid signaling is involved in nociception, neurotransmission, and neuroprotection.^169^ It is also engaged in learning, memory, motor, food intake, anxiety, pain perception, and fear memories.^170^ Cannabinoid receptor type 1 (CB-1) is the primary subtype in the central nervous system. In comparison, the CB-2 receptor is mainly found in immune tissues.^171^ Cannabinoid receptors in the presynaptic nerve terminals can be activated by endogenous lipid endocannabinoids 2-arachidonoylglycerol (2-AG) and N-arachidonoyl-ethanolamine (AEA; anandamide).^172^ 2-AG is a full agonist for cannabinoid receptors, while AEA is a weak partial agonist.^173^ Cannabinoid receptors can also be activated by phytocannabinoids such as Δ9-tetrahydrocannabinol and non-euphoric cannabidiol (CBD) extracted from cannabis.^174,175^

The CB-1 receptor is the dominant subtype in the brain.^176,177^ CB-1 receptor can be found in different neuronal types (e.g., GABAergic, glutamatergic, and serotonergic neurons) and controls cholinergic transmission.^178,179^ Exogenous administration of endocannabinoids protects neurons from β-amyloid (Aβ) neurodegeneration and apoptosis.^180^ Targeting cannabinoid receptors can improve spasticity (increase in muscle stiffness) and central neuropathic pain in patients with multiple sclerosis.^181^ Substantial reduction of CB-1 receptor in lateral globus pallidus and substantia nigra pars reticulata is associated with neurodegeneration in Huntington’s disease.^182,183^ Genetic polymorphisms on the CB-1 receptor are a risk factor for schizophrenia. CBD treatment is effective for neuroinflammatory-derived conditions such as epilepsy and anxiety.^184^

The pathological functions of the CB-2 receptor in inflammatory conditions (e.g., Alzheimer’s disease, Parkinson’s disease, multiple sclerosis, stress response, and depression) are under active investigation.^185,186^ Inflammation is a driving factor of depression and could counter the effects of antidepressant therapies.^187^ CB-2 receptor-overexpressing mice showed a significant reduction in depressive-related behaviors.^188^ In contrast, pro-inflammatory chemokines and cytokines are markedly reduced in the brain of CB-2 receptor-deficient mice.^189^ CB-2 receptor can suppress microglial activation and prevent pro-inflammatory mediators release.^190,191^ In bipolar disorder, a neuropsychiatric disorder presenting with mood fluctuation, selective activation of the CB-2 receptor can stabilize mood and reduce mood swings.^192^

#### Other receptors for endogenous cannabinoids: GPR12, GPR18, and GPR55

GPR12 is phylogenetically related to the cannabinoid (CB-1 and CB-2) receptors.^193^ GPR12 is a constitutively active receptor.^194^ Apart from cannabidiol, lysophospholipid sphingosine 1-phosphate and phingosyl-phosphorylcholine are potential endogenous ligands for GPR12.^193,195^ GPR12 expressed mainly in the central nervous system (frontal cortex, piriform cortex, thalamus, hypothalamus, hippocampus, amygdala, and olfactory bulb).^196^ In mice, GPR12 expresses in the area controlling emotion and metabolism.^195^ GPR12 promotes neurite outgrowth by activating ERK1/2 signaling.^197^ Other functions include pain control, neurite outgrowth, and regeneration.^193^ SNP microarray-based genome-wide association study reveals a close association between GPR12 and antipsychotic response in schizophrenia treatment.^198^

GPR18 and GPR55 also act as receptors for endogenous cannabinoids 2-AG and AEA.^199,200^ GPR18 and GPR55 exhibit high structural similarity.^201^ GPR18 regulates polymorphonuclear cell infiltration and protects organs from acute immune responses.^202^ It has been shown that GPR18 could interact with the CB-2 receptor in activated microglia of Alzheimer’s disease model;^203^ GPR55 expresses predominantly in the brain.^204^ The receptor can be activated by endocannabinoids, phytocannabinoids, synthetic cannabinoid ligands, and lysophosphatidylinositol.^205^ GPR55 antagonist exhibits anti-inflammatory functions by modulating GPR55-expressing immune cells such as monocytes and microglia.^206^ Given the high expression of GPR55 in the striatum, GPR55 signaling is suspected to be involved in motor impairment in Parkinson’s disease.^207^

#### Dopamine receptor

Dopamine is a catecholamine neurotransmitter in the brain. Dopamine/ dopamine receptors are crucial for motor function, cognition, learning, and memory.^208^ There are two receptor subtypes: D1-like (D1 and D5) and D2-like (D2, D3, and D4).^209^ D1 and D2 receptors are the most abundantly expressed dopamine receptor subtypes in the brain.^210^

D1 and D2 receptors are significantly reduced in asymptomatic Huntington’s disease patients.^12^ In the early stage of Huntington’s disease, dopamine signaling is associated with the development of dance-like movements (chorea). Clinical studies show that dopamine receptor blockers or depleting agents control motor dysregulation, especially chorea.^211^ In the late stage, however, a remarkable reduction in dopamine/ dopamine metabolite level is observed.^212^ The D1 receptor is remarkably reduced in patients presenting mild to moderate functional impairment.^213^ It is noted that targeting the dopaminergic signaling cascade might lead to rapid cognitive decline in Huntington’s disease patients.^214^

Disturbances in the dopaminergic system are frequently observed in other neurodegeneration disorders, including Alzheimer’s disease, Parkinson’s disease, and multiple sclerosis.^215^ Reduced dopamine receptors are correlated with the progression of Alzheimer’s disease;^216^ Loss of dopaminergic neurons is a hallmark feature of Parkinson’s disease. Activating D2-like receptors (D2/3 receptors) or increasing circulating dopamine are effective treatment strategies for symptomatic Parkinson’s disease;^217^ Dopamine dysregulation contributes to the demyelinating process (resulting from autoimmune attack) in multiple sclerosis.^218^ Dopamine can modulate pro-inflammatory cytokines secretion in T helper Th17 cells in uncontrolled neuroinflammatory responses.^219,220^

The development of β-arrestin-biased modulators might improve treatment outcomes and avoid side effects. Dopamine receptor agonist exhibits mild to serious side effects.^221^ This is partly caused by the activation of both G proteins and the β-arrestin signaling cascade.^222,223^ Many antipsychotics could interfere with dopamine-dependent β-arrestin 2 recruitment.^83^ Selective activating the D2 receptor-β-arrestin pathway with biased agonist is beneficial to correct dopamine signaling in schizophrenia.^224^

#### Histamine receptor

Histamine is an inflammatory biogenic amine synthesized from L-histidine. Histamine stimulates peripheral immune cells to release pro-inflammatory cytokines. In the central nervous system, histamine signaling in the tuberomammillary nucleus (TMN) controls sleep-wake, circadian and feeding rhythms.^225^ Elevated histamine increases blood-brain barrier permeability, allowing peripheral immune cells to enter and act on brain parenchyma.^226^

Four different histamine (H1–H4) receptors are reported.^227^ H1 and H2 receptors are expressed in the brain, central nervous system, and peripheral tissues.^225^ H1 receptor activation promotes neuron differentiation. In contrast, H2 receptor activation induces neural stem cell proliferation.^228^ H3 receptor is localized in the brain.^229^ H3 receptor is an important therapeutic target for cognitive disorders.^230^ The neurological function of the H4 receptor remains unclear.^229^ H4 receptor can be detected in the non-neuronal cells of the brain.^229^ H4 receptor activation is involved in the inflammatory responses regulated by mast cells, eosinophils, and T cells.^229^ Histamine acts on H1 and H3 receptors to control normal sleep/wake behavior.^231^

Alterations in histamine signaling are found in both neurodegenerative and psychiatric disorders.^230^ Due to structural similarity, H1 and H4 receptors are suggested to have cross-functional impacts on disease development. Positron emission tomography results show that reduced H1 or H4 receptor is present in a subgroup of Alzheimer’s disease, schizophrenic and depressed patients.^232–234^ The role of histamine signaling in Alzheimer’s disease remains controversial due to the conflicting results on histamine levels.^232^ H1 receptor upregulation is associated with myelin damage mediated by focal lymphocytes in multiple sclerosis.^235^ Targeting the H3 receptor with selective antagonists could stimulate the release of crucial neurotransmitters, including acetylcholine, dopamine, norepinephrine, and histamine.^236^ H4 receptor is involved in M1-activated microglia cells (primary inflammatory cells in the brain) driven neuroinflammation. Attenuating H4 receptor signaling is beneficial in controlling inflammation propagation in Parkinson’s disease.^237^

#### Melanin-concentrating hormone receptor

Melanin-concentrating hormone (MCH) is the pro-melanin expressed by the central nervous system.^238^ MCH is well documented for its function in controlling motivated behaviours, including feeding and drinking.^239^ Later studies suggest that MCH promotes non-REM sleep and modulates energy homeostasis.^240,241^ MCH receptor 1 is a stress modulator regulating fear and anxiety processes.^242^ MCH receptor 1-signaling is responsive to physiological- or neurochemical-controlling stress and affective states in genetically knockout models.^243^ MCH is associated with behavioural disorders and depressive symptoms observed in Huntington’s disease patients.^244^ Animals without MCH receptor expression exhibit schizophrenia-like phenotypes.^245^

#### Melatonin receptor

Melatonin (MT) or *N*-acetyl-5-methoxytryptamine is a neuroendocrine hormone produced by the pineal gland. MT is a regulator of the circadian rhythm (sleep-wake cycle). Melatonin is converted from tryptophan/ serotonin in the pinealocytes. Melatonin also functions as an antioxidant to protect tissues from free radical damage.^246^ The antioxidant activity of melatonin is essential in tissue (such as the brain) with high reactive oxygen species (ROS) resulting from oxygen consumption.^247^ Peripheral tissues, such as the gut and skin, could also secrete melatonin.^248^ Melatonin secretion is suppressed by daylight through the retino‐hypothalamic tract and reaches a peak at night. Rhythmic nocturnal secretion (secreted in the dark) allows melatonin to distributes throughout the body via circulation.

Circadian rhythm dysregulation is a common symptom presented by patients with neurodegenerative disease due to functional impairment of the retina-suprachiasmatic nucleus (SCN)-pineal axis.^249^ In Alzheimer’s disease, melatonin and MT1 receptor level in SCN and cortex diminishes remarkably.^250,251^ Pathological α-synuclein aggregation (a stepwise aggregation of presynaptic neuronal protein observed during Parkinson’s disease development) is reduced in animal models subjected to melatonin treatment.^252,253^ In multiple sclerosis and amyotrophic lateral sclerosis, melatonin demonstrates anti-apoptotic functions and offers neural protection from oxidative damage.^254,255^

Dysregulation in MT1/2 receptor signaling contributes to the pathological development of anxiety, sleep disorders (insomnia), and depression.^256–258^ In a post-mortem study on depressed patients, hypothalamic MT1 expression increased in the hypothalamic suprachiasmatic nucleus and is correlated with disease duration.^259^ Melatonin treatment can alleviate symptoms of psychiatric disorders with few side effects (even at high dosages).^260^ Exogenous melatonin may also be administered to control anxiety.^261^ Melatonin is an effective medication for sleep disturbances in depression.^262^ However, no solid empirical evidence supports melatonin or melatonin receptor agonists as the cure for depression. The use of melatonin to normalize the disrupted circadian cycle might not be sufficient to alleviate depression.

#### Sphingosine 1-phosphate (S1P) receptor

S1P is an active lysophospholipid. S1P exerts its biological functions through S1P receptor 1-5.^263^ S1P/S1P receptor controls angiogenesis, chemotaxis, and egress of lymphocytes (from bone marrow, thymus, and lymphoid tissues).^263^ S1P receptor-expressing immune cells in lymphoid tissues are attracted by the high S1P level in the bloodstream.^264^ S1P receptors on immune cells are inactivated in peripheral blood by receptor internalization.^264^ S1PR1 can be found in B, T, and dendritic cells.^263^ During inflammation, S1PR1 on immune cells is upregulated.^265^ S1PR1 enhances inflammation by activating neuroglia/microglia (immune cells orchestrating inflammatory response in the central nervous system).^266^ S1PR1 might contribute to the development of multiple sclerosis by promoting chronic and acute inflammation.^263,267^ Unlike S1PR1, S1PR5 is mainly detected in natural killer and dendritic cells.^268^ S1PR5 expression on natural killer cells is critical for its egress from lymph nodes and bone marrow.^269^ Hence, targeting S1P receptors might protect the brain from immune attacks by limiting lymphocytes from passing through the blood-brain barrier in multiple sclerosis.^266^

#### Opioid receptor

The opioid receptor family is composed of delta (δ)-opioid receptor (DOR), kappa (κ)-opioid receptor (KOR), mu (μ)-opioid receptor (MOR), and nociceptin receptor. Opioid receptor recognizes a variety of endogenous neuropeptides, including enkephalins, endorphins, and dynorphins.^270^ The endogenous opioids are one of the neuromodulators produced by the body to attenuate stressful states. Opioid receptor in the central and peripheral nervous system regulates stress and pain responses.^271^ locus coeruleus (LC) in the brain is the stress-integrating site. The opioid receptor can sensitize neurons in LC to corticotropin-releasing factor (CRF), a potent psychological mediator regulating stress-induced behaviors.^272^ Chronic or persistent acute stress can alter LC functions.^273^ Hyperactive LC is associated with psychiatric disorders.^274^ Dysregulation of the opioid receptors affects emotion processing in patients with major depressive disorders.^275^ Opioid receptor levels are related to neurocognitive deficits.^276^ Elevated opioid receptors level might elicit symptoms of schizophrenia resulting in treatment resistance.^276^

δ-opioid receptor and mu-opioid receptor exhibit opposite functions in the pathogenesis of Alzheimer’s disease. δ-opioid receptor agonist reduces expression of β-site APP cleaving enzyme 1 (BACE1), which cleaves amyloid precursor protein to initiate Aβ peptide production in PC12 cells (harbouring mimicked injury of Alzheimer’s disease).^277,278^ On the contrary, knocking down δ-opioid receptor increases BACE1 expression, leading to high production of Aβ42, the essential pathogenic Aβ peptides in Alzheimer’s disease with 42 amino acids.^278^ For the μ-opioid receptor, it is noted that agonist-induced receptor activation enhances BACE1 and Aβ42 expression.^278^ Hence, targeting δ-opioid/μ-opioid receptor signaling might benefit Alzheimer’s disease treatment; Parkinson’s disease patients have reduced brain kappa-opioid receptor levels.^279^ Activating κ-opioid receptor ameliorates Parkinsonian behaviours and restores locomotor in marmoset with Parkinsonism.^280^ In addition, κ-opioid receptor agonists can alleviate dyskinesia behaviour derived from L-DOPA in Parkinson’s disease rats.^281^

#### Serotonin receptor

Dysregulation of serotonin (5-hydroxytryptamine, 5-HT) receptors is observed in nearly all neurodegenerative and psychiatric disorders.^282,283^ 5-HT receptors 1 and 2 are the most intensively studied drug targets. The receptors have various effects with multiple subtypes and alternative splice variants. 5-HT1 and 5-HT2 receptors have different expression patterns in the brain with similar or opposite functions.^284^ 5-HT1 receptor has 5 subtypes: 5-HT1A, 5-HT1B, 5-HT1D, 5-HT1E and 5-HT1F receptors. 5-HT1A receptor can be found in both serotonin neurons and non-serotonin neurons.^285^ 5-HT1A receptor is associated with anxiety and mental traits in transgenic mice.^285^ Partial agonists targeting the 5-HT1A receptor are suggested to be useful in controlling alcohol abuse.^286^ Anterior cingulate cortex (ACC) is a brain region regulating emotion regulation, pain perception, and cognitive control.^287^ Patients with bipolar disorder, major depressive disorder, and schizophrenia have higher 5-HT1B receptor expression in the outer ACC layers compared to the inner ACC layers;^288^ 5-HT2 receptor has 3 subtypes: 5-HT2A, 5-HT2B, and 5-HT2C receptors. 5-HT2 receptors are implicated in various neuropsychiatric phenotypes, including schizophrenia, attention deficit hyperactivity disorder, affective disorders, eating disorders, anxiety disorders, obsessive-compulsive disorder, suicide, and Alzheimer’s disease.^289^

### Class C (glutamate)

#### Structural insights

Class C GPCRs are distinguished from other classes of GPCRs by two unique features. First, the orthosteric ligand binding pocket is located in the large extracellular venus flytrap domain (VFT). VFT is connected to the transmembrane helix via the cysteine-rich domain (CRD) (Fig. 4c). Among class C GPCRs, only the GABAB receptor lacks CRD; Second, class C GPCR forms hetero- or homo-dimers at physiological conditions.^290–294^ VFT domain forms an asymmetric dimer interface to facilitate dimer formation. Ligand engagement at either subunit is sufficient to activate the receptor.^291,294,295^ The surface interface between dimers is the potential binding site for the therapeutic modulator.^292^ The conformation rearrangement between ICL2 and ICL3, and C-terminus contributes to receptor activation.^296–298^

#### γ-aminobutyric acid B receptor

γ-aminobutyric acid B (GABAB) is an inhibitory neurotransmitter. GABAB receptor is a heterodimer consisting of two subunits, GABAB1 and GABAB2. GABAB1 expression is reduced in the brain of Alzheimer’s disease patients. The GABAB1 protein level is negatively associated with the neurofibrillary tangle.^299^ Results from a genome-wide association study (GWAS) show that GABAB1 SNPs are a risk factor for schizophrenia.^300^ GABAB2 SNPs are correlated with the development of Huntington’s disease.^301^ Activating GABAB receptor can ameliorate motor impairment and reduces inflammation/ oxidative damage in Parkinson’s disease models.^302^

#### Metabotropic glutamate receptors

The excitatory neurotransmitter glutamate mediates neuronal excitability via metabotropic glutamate receptors (mGluRs). Functional mGluR is a homodimeric receptor consisting of 8 members (mGluR1-8).^303^ Dysregulation of mGluR signaling pathways is observed in both neurodegenerative and psychiatric disorders.^304^

Group I mGluR composes of mGluR1 and mGluR5. mGluR1 localizes in the hippocampus, hypothalamus, periaqueductal gray, and amygdala, which are associated with anxiety.^305^ mGluR5 activity is linked to the cognitive symptoms of Alzheimer’s disease.^306–308^ Deleting mGluR5 improved spatial learning impairment and decreased Aβ oligomers in Alzheimer’s disease models.^309^ Interaction between mGluR5 and cellular prion protein could also play a part in the pathogenesis of Alzheimer’s disease.^310,311^ Activating mGluR5 promotes striatal neuron survival in Huntington’s disease models.^312,313^ mGluR5 knockout mice exhibit obvious schizophrenia symptoms, including reduced spatial memory and reduced sensorimotor gating.^314^

Group II mGluR consists of mGluR2 and mGluR3. Activating mGluR2 and mGluR3 can control panic-like behaviors and ameliorates acute stress responses in the anxiety model.^315^ Mutant huntingtin in Huntington’s disease is toxic to neurons.^313^ In the mouse model, activating mGluR2 and mGluR3 could enhance limb coordination by attenuating the generation of huntingtin aggregate.^316^ mGluR2 and mGluR3 demonstrate protective effects on the nigrostriatal system, which restores functional deficits in Parkinson’s disease rat model.^317,318^ Overexpression of mGluR2 in the neocortical layers, cerebellum, striatum, hippocampus, and thalamus/hypothalamus could build up glutamate-mediated excitotoxicity and promote Huntington’s disease progression.^319–322^

Group III mGluR includes mGluR4/6/7/8. mGluR4 activation ameliorates locomotion disorder in Parkinson’s disease rats.^323^ mGluR7/8 are associated with the anxiety-related phenotype.^324,325^ SNPs in mGluR7/8 are correlated to the susceptibility of schizophrenia.^326–329^

#### GPCR dimers

GPCRs can function in homodimeric or heterodimeric forms.^330,331^ The receptor complex consists of one GPCR dimer with two orthosteric binding sites and a heterotrimeric G protein.^332^ GPCR dimer exhibits different biochemical properties compared to the individual receptor. Activation of either one of the receptors is sufficient to promote dimer formation.^333^ Dimeric GPCR has a different ligand binding affinity as compared to the monomer.^331^ Receptor dimerization affects receptor trafficking in agonist-induced GPCR endocytosis.^330^ Closely related GPCR subtypes are more efficient in forming heteromers.^334^ Here, we focused on discussing two physiologically existing GPCR heterodimers (A2AR-D2R and mGluR2-5-HT2A).

Adenosine 2A receptor-dopamine D2 receptor (A2AR-D2R) heterodimer is located in the ventral striato-pallidal GABA neurons.^335,336^ A2AR-D2R heterodimer attracts attention in the field of Parkinson’s disease medication as ligands for A2AR can modulate dopamine signaling in *Parkinson’s* disease. Co-administration of dopamine precursor L-DOPA (L-3,4-dihydroxyphenylalanine) and dopamine receptor agonists could improve mobility in Parkinson’s disease.^141^ It has been shown that adenosine antagonists such as caffeine could enhance dopamine agonist action in Parkinson’s disease treatment.^337^ A2AR activation can suppress D2R-mediated Gi/o signaling.^335^ Stimulating A2AR with adenosine A2AR agonist in the nucleus accumbens produces behavioural effects similar to local dopamine depletion.^338^ Thus, the action of A2AR modulators should be considered in the drug design for Parkinson’s disease.

Serotonin type A 5-HT2A receptor and type C metabotropic glutamate 2 (mGlu2) receptor regulates psychoactive behavior in schizophrenia.^339,340^ 5-HT2A receptor is a Gq-coupled receptor, while mGluR2 receptor signals through Gi.^341^ 5-HT2A receptor is upregulated in the frontal cortex of schizophrenic subjects compared with normal subjects. In contrast, the expression level of mGluR2 is decreased.^341^ Balance between Gq and Gi is a predictive indicator of antipsychotic drug properties.^342^ 5-HT2A receptor and mGluR2 can form stable complexes in physiological conditions which regulate Gq-Gi balance cooperatively.^343^ mGluR2 agonist reduces 5-HT2A receptor/Gq signaling in the frontal cortex of schizophrenic subjects.^341^ mGluR2 agonist can downregulate 5-HT2A receptor expression.^344^ On the contrary, it has been shown that the 5-HT2A receptor controls mGluR2 expression at the epigenetic level in the frontal cortex.^342^ Although the 5-HT2A receptor and mGluR2 regulate the activity of each other remains elusive, interrupting the functional crosstalk in the 5-HT2A receptor/ mGluR2 complex is a putative approach in schizophrenia treatment.^345^

## Therapeutic development

**Fig. 5:**
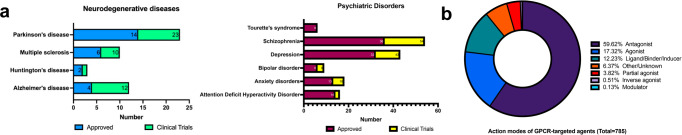
GPCRs-targeting drugs for neurodegenerative diseases and psychiatric disorders. **a** Numbers of compounds approved for clinical use or under clinical trials. **b** Summary of the action modes of GPCR-targeted agents for treatment of neuropsychiatric diseases

**Fig. 6:**
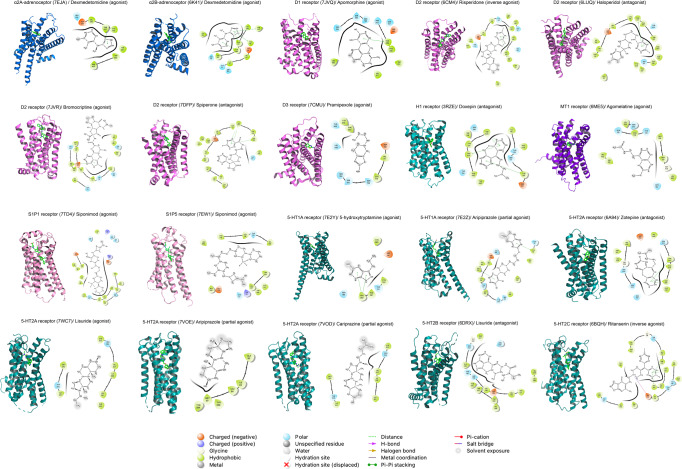
Interactions between neuropsychiatric drugs with key residues in the orthosteric ligand binding pocket of GPCRs (e.g., adrenoceptors, dopamine receptors, histamine receptors, melatonin receptors, S1P1/5 receptors, and serotonin receptor). The small molecules regulate GPCR activity by stabilizing receptors at unique conformational state

The small molecules regulate GPCR activity by stabilizing receptors at unique conformational state (Fig. 5). To explore the GPCRs-based therapeutic strategies against neuropsychiatric disorders, we examined the clinically approved drugs (Fig. 5a) and compounds being tested in different stages of clinical trials (Fig. 5b) in the DrugBank database (https://go.drugbank.com/). In total, 92 drugs are being approved (Table 2). Forty-one candidates are undergoing clinical trials (Table 3). Selected receptors/drugs interaction are shown in Fig. 6.

**Table 2:** Approved drugs for neuropsychiatric disorders

Drug	Structure	Indication	GPCRs	Mechanism	*K*_i_ (nM)	Reference
Donepezil		Alzheimer’s disease	5-HT-2A	Inducer	/	^605^
Memantine		Alzheimer’s disease	5-HT-3A	Antagonist	/	^606,607^
			DRD2	Antagonist; Agonist	/	
Olanzapine		Schizophrenia; depression	5-HT-2C	Antagonist	2.8	^608–619^
			HH1R	Antagonist	0.087	
			DRD2	Antagonist	2.1	
			DRD3	Antagonist	39	
			DRD4	Antagonist	28	
			DRD5	Antagonist	74	
			ADRA1A	Antagonist	/	
			ADRA1B	Antagonist	/	
			5-HT-2A	Antagonist	1.48	
			5-HT-3A	Antagonist	/	
			5-HT-6	Antagonist	6	
			DRD1	Antagonist	10	
			CHRM1	Antagonist	2	
			CHRM2	Antagonist	36	
			CHRM3	Antagonist	13	
			CHRM4	Antagonist	10	
Thioridazine		Alzheimer’s disease; schizophrenia	DRD1	Antagonist	100	^620–623^
			DRD2	Antagonist	27	
			5-HT-2A	Antagonist	10	
			ADRA1B	Antagonist	/	
			ADRA1A	Antagonist	/	
Trazodone		Alzheimer’s disease; schizophrenia; depression; anxiety disorders	5-HT-2A	Antagonist	44.67	^624,625^
			5-HT-2C	Agonist	25	
			5-HT-1A	Antagonist; Partial agonist	96	
			HH1R	Antagonist	1100	
			ADRA1A	Antagonist	/	
			ADRA2A	Antagonist	106	
			5-HT-1C	Antagonist; Partial agonist	/	
Amantadine		Parkinson’s disease	DRD2	Agonist	/	/
Apomorphine		Parkinson’s disease	DRD4	Agonist	8.9	^364,626,627^
			DRD2	Agonist	0.62	
			DRD3	Agonist	2.6	
			DRD5	Agonist	14.79	
			DRD1	Agonist	4.6	
			ADRA2C	Agonist	36.31	
			ADRA2B	Agonist	66.07	
			5-HT-1A	Agonist	296	
			5-HT-2A	Agonist	120.23	
			5-HT-2B	Agonist	/	
			5-HT-2C	Agonist	102.33	
			ADRA2A	Agonist	141.25	
			5-HT-1D	Agonist	1230.27	
			5-HT-1B	Agonist	2951.21	
Benzatropine		Parkinson’s disease	CHRM1	Antagonist	/	^628–630^
			HH1R	Antagonist	/	
Biperiden		Parkinson’s disease	CHRM1	Antagonist	0.48	^630^
Bromocriptine		Parkinson’s disease	DRD2	Agonist	10	^364,631–633^
			DRD3	Agonist	87	
			5-HT-1D	Agonist	10.72	
			ADRA2A	Agonist	10.96	
			5-HT-1A	Agonist	12.88	
			ADRA2C	Agonist	28.18	
			ADRA2B	Agonist	34.67	
			5-HT-2B	Agonist	/	
			DRD4	Antagonist	/	
			5-HT-2A	Agonist	107.15	
			5-HT-1B	Agonist	354.81	
			5-HT-2C	Agonist	741.31	
			DRD5	Agonist	454	
			DRD1	Agonist	672	
			ADRA1A	Antagonist; Agonist	/	
			ADRA1B	Antagonist; Agonist	1.38	
			ADRA1D	Agonist	1.12	
			5-HT-7	Antagonist	/	
Droxidopa		Parkinson’s disease	ADRA1A	Agonist	/	^634,635^
			ADRA1B	Agonist	/	
			ADRA1D	Agonist	/	
			ADRA2A	Agonist	/	
			ADRA2B	Agonist	/	
			ADRA2C	Agonist	/	
			ADRB1	Agonist	/	
			ADRB2	Agonist	/	
			ADRB3	Agonist	/	
Istradefylline		Parkinson’s disease	ADORA2A	Antagonist	/	^365,636^
			ADORA1	Antagonist	/	
Levodopa		Parkinson’s disease	DRD1	Agonist	/	^637–640^
			DRD2	Agonist	/	
			DRD3	Agonist	/	
			DRD4	Agonist	/	
			DRD5	Agonist	/	
Pergolide		Parkinson’s disease	DRD4	Agonist		^364,620,632,640–645^
			DRD5	Agonist		
			DRD1	Agonist	2020	
			DRD3	Agonist	4	
			DRD2	Agonist	4	
			5-HT-1A	Agonist	1.8	
			5-HT-2B	Agonist	/	
			5-HT-2A	Agonist	/	
			5-HT-1D	Agonist	/	
			5-HT-1B	Agonist	/	
			5-HT-2C	Agonist	/	
			ADRA2	Agonist	/	
			ADRA1A	Agonist	/	
			ADRA1B	Agonist	/	
			ADRA1D	Agonist	/	
Pramipexole		Parkinson’s disease	DRD3	Agonist	0.87	^370,646,647^
			DRD2	Agonist	21	
			DRD4	Agonist	8.1	
			5-HT-1A	Agonist	/	
			ADRA2A	Agonist	/	
Quetiapine		Parkinson’s disease; bipolar disorder; schizophrenia	5-HT-2A	Antagonist	31	^385,436,437,619,648–651^
			DRD2	Antagonist	69	
			5-HT-1A	Antagonist; Partial agonist	125	
			5-HT-1B	Ligand	2050	
			5-HT-1D	Ligand	560	
			5-HT-1E	Ligand	1250	
			5-HT-2C	Antagonist	615	
			5-HT-3A	Ligand	/	
			5-HT-6	Antagonist	33	
			5-HT-7	Ligand	/	
			DRD5	Ligand	1513	
			DRD3	Ligand	320	
			DRD4	Ligand	1600	
			HH1R	Antagonist	2.2	
			ADRA1	Antagonist	/	
			ADRA2A	Antagonist	80	
			ADRA2B	Antagonist	90	
			ADRA2C	Antagonist	28.7	
			CHRM1	Antagonist	56	
			CHRM2	Ligand	630	
			CHRM3	Antagonist	705	
			CHRM4	Ligand	225	
			CHRM5	Ligand	/	
			DRD1	Antagonist	390	
Ropinirole		Parkinson’s disease	DRD2	Agonist	7.2	^364,370,645,652^
			DRD4	Agonist	/	
			DRD3	Agonist	19	
			ADRA1	Antagonist	/	
Rotigotine		Parkinson’s disease	DRD2	Agonist	0.06	^364,653^
			DRD3	Agonist	4	
			DRD5	Agonist	986	
			DRD1	Agonist	2172	
			DRD4	Agonist	55	
			ADRA2B	Antagonist	/	
			5-HT-1A	Agonist	/	
lisuride		Parkinson’s disease	DRD2	Agonist	0.5	^364,629,633^
			DRD1	Antagonist	77	
			DRD3	Agonist	1.7	
			DRD4	Agonist	/	
			DRD5	Antagonist	/	
			ADRA2B	/	/	
			ADRA2A	/	/	
			ADRA2C	/	/	
			5-HT-1A	Agonist	0.4	
			5-HT-2A	Agonist	6918.31	
			5-HT-2C	Agonist	/	
			5-HT-1D	Agonist	/	
			5-HT-2B	Antagonist		
			5-HT-1B	Agonist		
			5-HT-7	Inactivating antagonist		
Baclofen		Multiple sclerosis	GABBR2	Agonist	/	^654,655^
			CXC-R4	Allosteric; modulator	/	
			GABBR1	Agonist	/	
Cannabidiol		Multiple sclerosis	CB-R	Antagonist	/	^656–658^
			CB-2	Antagonist	/	
			GPR12	Inverse agonist	/	
			GPR18	/	/	
			GPR55	Antagonist	/	
			5-HT-1A	Agonist	/	
			5-HT-2A	Agonist	/	
			DOR-1	/	/	
			MOR-1	/	/	
			5-HT-3A	Antagonist	/	
			ADORA1	Activator	/	
Modafinil		Multiple sclerosis; attention deficit hyperactivity disorder	ADRA1B	Partial agonist	/	^659^
Ozanimod		Multiple sclerosis	S1PR1	Agonist	/	^660^
			S1PR5	Agonist	/	
Siponimod		Multiple sclerosis	S1PR1	Agonist	/	^661^
			S1PR5	Agonist	/	
Fingolimod		Multiple sclerosis	S1PR5	Agonist	/	^662,663^
			S1PR1	Agonist	/	
			S1PR3	Agonist	/	
			S1PR4	Agonist	/	
Fluphenazine		Tourette’s disorder; depression	DRD2	Antagonist	1.44	^629,664,665^
			DRD1	Antagonist	7	
			5-HT-2A	Antagonist	3.2	
			5-HT-2C	Antagonist	579	
Haloperidol		Huntington’s disease; schizophrenia	5-HT-2C	/	/	^613,619,623,666–669^
			5-HT-2A	Antagonist	25	
			DRD1	Antagonist	6.17	
			DRD2	Antagonist	0.12	
			DRD3	Inverse agonist	2	
			HH1R	/	/	
			CHRM3	/	/	
			ADRA1A	/	/	
			ADRA2A	/	/	
			ADRA2B	/	/	
			ADRA2C	/	/	
			5-HT-1A	/	/	
			5-HT-6	/	/	
			5-HT-7	/	/	
			MCHR1	/	/	
Tetrabenazine		Huntington’s disease	DRD2	Inhibitor	/	/
Amitriptyline		Schizophrenia; depression; attention deficit hyperactivity disorder;	5-HT-2A	Antagonist	/	^407,670–681^
			5-HT-1A	Inhibitor; Inducer	450	
			DOR-1	Agonist	/	
			KOR-1	Agonist	/	
			ADRA1A	Antagonist; Inhibitor	/	
			ADRA1D	Antagonist	/	
			ADRA2A	Antagonist; Agonist	114	
			HH1R	Antagonist	0.67	
			HH2R	Blocker	/	
			HH4R	Binder	33.6	
			5-HT-2C	Antagonist	18	
			ADRA1B	Antagonist	/	
			5-HT-7	Antagonist	/	
			5-HT-1D	Binder	/	
			MOR-1	Binder	/	
			5-HT-1B	Binder	/	
			5-HT-6	Antagonist	65	
			5-HT-1C	Antagonist	/	
			CHRM	Ligand	/	
Aripiprazole		Schizophrenia; Tourette’s disorder	DRD2	Antagonist; Partial agonist	0.2	^386^
			5-HT-2A	Antagonist; Partial agonist	0.8	
			5-HT-1A	Partial agonist	5.6	
			ADRA1A	Antagonist	/	
			ADRA1B	Antagonist	34.8	
			DRD3	Antagonist; Partial agonist	3.3	
			5-HT-1D	Antagonist; Partial agonist	68	
			5-HT-7	Antagonist; Partial agonist	14	
			ADRA2A	Antagonist	74	
			ADRA2C	Antagonist; Other/unknown	37	
			HH1R	Antagonist	25.1	
			5-HT-1B	Antagonist; Ligand	830	
			5-HT-2C	Antagonist; Partial agonist	22	
			5-HT-3A	Antagonist	/	
			5-HT-6	Antagonist	90	
			DRD1	Antagonist; Partial agonist; Ligand	1960	
			DRD4	Antagonist; Partial agonist	168	
			ADRA2B	Antagonist; Ligand	102	
			5-HT-1E	Antagonist; Ligand	8000	
			DRD5	Antagonist; Partial agonist; Ligand	2590	
			5-HT-2B	Inverse agonist	/	
			5-HT-5A	Ligand	/	
			ADRB1	Ligand	/	
			ADRB2	Ligand	/	
			HH2R	Ligand	/	
			HH3R	Ligand	/	
			HH4R	Ligand	/	
			CHRM1	Ligand	/	
			CHRM2	Ligand	/	
			CHRM3	Ligand	/	
			CHRM4	Ligand	/	
			CHRM5	Ligand	/	
			KOR-1	Ligand	/	
			MOR-1	Ligand	/	
			DOR-1	Ligand	/	
Aripiprazole lauroxil		Schizophrenia	DRD2	Partial agonist	/	^619,682,683^
			5-HT-1A	Partial agonist	/	
			5-HT-2A	Antagonist	/	
			5-HT-1B	/	/	
			5-HT-1D	/	/	
			5-HT-1E	/	/	
			DRD1	/	/	
			DRD5	/	/	
			DRD3	/	/	
			DRD4	/	/	
			5-HT-2C	/	/	
			5-HT-3A	/	/	
			5-HT-6	/	/	
			5-HT-7	/	/	
			HH1R	Antagonist	/	
			ADRA1A	Antagonist	/	
			ADRA1B	Antagonist	/	
			ADRA2A	/	/	
			ADRA2B	/	/	
			ADRA2C	/	/	
			CHRM1	/	/	
			CHRM2	/	/	
			CHRM3	/	/	
			CHRM4	/	/	
			CHRM5	/	/	
Asenapine		Schizophrenia	ADRA1A	Antagonist	/	^684^
			ADRA2A	Antagonist	/	
			ADRA2B	Antagonist	/	
			ADRA2C	Antagonist	/	
			ADRB1	Antagonist	/	
			ADRB2	Antagonist	/	
			DRD4	Antagonist	/	
			DRD3	Antagonist	/	
			5-HT-1A	Antagonist	/	
			5-HT-1B	Antagonist	/	
			5-HT-2B	Antagonist	/	
			5-HT-2A	Antagonist	/	
			5-HT-2C	Antagonist	/	
			5-HT-2B	Antagonist	/	
			5-HT-5A	Antagonist	/	
			5-HT-6	Antagonist	/	
			5-HT-7	Antagonist	/	
			HH1R	Antagonist	/	
			HH2R	Antagonist	/	
			DRD1	Antagonist	/	
			DRD2	Antagonist	/	
Brexpiprazole		Schizophrenia; major depressive disorder (MDD)	5-HT-1A	Agonist; Partial agonist	/	^389,390^
			DRD2	Agonist; Partial agonist	/	
			5-HT-2A	Antagonist	/	
			ADRA2C	Antagonist	/	
			ADRA1B	Antagonist	/	
Cariprazine		Schizophrenia	DRD2	Partial agonist	/	^685,686^
			DRD3	Partial agonist	/	
			ADRA1A	Antagonist	/	
			5-HT-1A	Partial agonist	/	
			5-HT-2A	Antagonist	/	
			5-HT-2B	Antagonist	/	
			5-HT-2C	Antagonist	/	
			HH1R	Antagonist	/	
Chlorpromazine		Schizophrenia	DRD2	Antagonist	1.2	^622,687,688^
			DRD1	Antagonist	44	
			5-HT-1A	Antagonist	116.4	
			5-HT-2A	Antagonist	1.8	
			ADRA1A	Antagonist	/	
			ADRA1B	Antagonist	/	
			HH1R	Antagonist	3	
			DRD3	Inhibitor	3	
			DRD4	Antagonist	/	
			DRD5	Inhibitor	133	
			5-HT-2C	Binder	1.4	
			ADRA1	Inhibitor	/	
			ADRA2	Inhibitor	/	
			CHRM1	Antagonist	25	
			CHRM3	Antagonist	47	
			5-HT-6	Binder	4	
			5-HT-7	Binder	27	
			HH4R	Binder	50.2	
Chlorprothixene		Schizophrenia	HH1R	Antagonist	3.73	^689–693^
			DRD2	Antagonist	2.96	
			DRD1	Antagonist	18	
			DRD3	Antagonist	4.56	
			5-HT-2A	Antagonist		
			CHRM1	Antagonist	11	
			CHRM2	Antagonist	28	
			CHRM3	Antagonist	22	
			CHRM4	Antagonist	18	
			CHRM5	Antagonist	/	
			5-HT	Inhibitor	/	
Clozapine		Schizophrenia	DRD2	Antagonist	28	^608,609,611,613,619,667,694–706^
			5-HT-2A	Antagonist	1	
			5-HT-1A	Antagonist	101	
			5-HT-1B	Antagonist	390	
			5-HT-1D	Antagonist	130	
			5-HT-1E	Antagonist	430	
			5-HT-3A	Antagonist	/	
			5-HT-2C	Antagonist	1.8	
			5-HT-6	Antagonist	4	
			5-HT-7	Antagonist	9	
			DRD1	Antagonist	53	
			DRD3	Antagonist	88	
			DRD4	Antagonist	9	
			ADRA1A	Antagonist	/	
			ADRA1B	Antagonist	/	
			ADRA2A	Antagonist	15	
			ADRA2B	Antagonist	22	
			ADRA2C	Antagonist	2.9	
			CHRM1	Antagonist	0.98	
			CHRM2	Antagonist	9	
			CHRM3	Antagonist	7	
			CHRM4	Antagonist	6	
			CHRM5	Antagonist	/	
			HH1R	Antagonist	0.23	
			HH4R	Antagonist	11.9	
Dexmedetomidine		Schizophrenia; bipolar disorder	ADRA2A	Agonist	2.0417	^707^
Fluspirilene		Schizophrenia	DRD2	Antagonist	/	^630^
			5-HT-2A	Antagonist	9.5	/
Iloperidone		Schizophrenia	5-HT-2A	Antagonist	0.12	^708,709^
			DRD2	Antagonist	/	
			DRD1	Antagonist	216	
			DRD3	Antagonist	./	
			DRD4	Antagonist	/	
			5-HT-1A	Antagonist	33	
			5-HT-6	Antagonist	63.1	
			5-HT-7	Antagonist	/	
			ADRA1A	Antagonist	/	
			HH1R	Antagonist	12.3	
			ADRA2C	Antagonist	16.2	
Loxapine		Schizophrenia	5-HT-2A	Antagonist	2	^623,629,691,710–716^
			5-HT-2C	Antagonist	1.69	
			5-HT-1A	Binder	2456	
			5-HT-1B	Binder		
			5-HT-1D	Binder	/	
			5-HT-1E	Binder	/	
			5-HT-3A	Binder	/	
			5-HT-5A	Binder	/	
			5-HT-6	Binder	15	
			5-HT-7	Binder	/	
			ADRA1A	Binder	/	
			ADRA1B	Binder	/	
			ADRA2A	Binder	150.8	
			ADRA2B	Binder	107.6	
			ADRA2C	Binder	79.9	
			ADRB1	Binder	/	
			CHRM1	Binder	63.9	
			CHRM2	Binder	300	
			CHRM3	Binder	122	
			CHRM4	Binder	300	
			CHRM5	Binder	/	
			DRD1	Antagonist	/	
			DRD2	Antagonist	21	
			DRD3	Antagonist	22	
			DRD4	Antagonist	4.9	
			DRD5	Binder	/	
			HH1R	Binder	4.9	
			HH2R	Binder	/	
			HH4R	Binder	3981	
Lumateperone		Schizophrenia; depression, bipolar	5-HT-2A	Antagonist	/	^392^
			DRD2	Partial agonist	/	
			DRD1	/	/	
Lurasidone		Schizophrenia; depression, bipolar	5-HT-2A	Antagonist	/	^717,718^
			5-HT-1A	Antagonist	/	
			ADRA2C	Antagonist	/	
			5-HT-7	Antagonist	/	
			ADRA2A	Unknown	/	
			DRD2	Antagonist	/	
Methotrimeprazine		Schizophrenia; anxiety bipolar disorder (BD)	DRD2	Antagonist	/	^719^
			DRD1	Antagonist	/	
			DRD5	Antagonist	/	
			DRD3	Antagonist	/	
			DRD4	Antagonist	/	
			5-HT-2A	Antagonist	/	
			5-HT-2C	Antagonist	/	
			HH1R	Antagonist	/	
			CHRM1	Antagonist	/	
			CHRM2	Antagonist	/	
			CHRM3	Antagonist	/	
			CHRM4	Antagonist	/	
			CHRM5	Antagonist	/	
			ADRA1A	Antagonist	/	
			ADRA1B	Antagonist	/	
			ADRA1D	Antagonist	/	
			ADRA2A	Antagonist	/	
			ADRA2B	Antagonist	/	
			ADRA2C	Antagonist	/	
Paliperidone		Schizophrenia	5-HT-2A	Antagonist	0.43	^439,608,700,720–722^
			5-HT-1A	Antagonist	480	
			5-HT-2C	Antagonist	/	
			5-HT-1D	Antagonist	19	
			5-HT-7	Inactivating antagonist	/	
			HH1R	Antagonist	3.4	
			ADRA1A	Antagonist	/	
			ADRA2A	Antagonist	30	
			ADRA1B	Antagonist	/	
			ADRA2B	Antagonist	9.4	
			ADRA2C	Agonist	11	
			DRD1	Antagonist	/	
			DRD2	Antagonist	/	
			DRD3	Antagonist	/	
Prochlorperazine		Schizophrenia	DRD2	Antagonist	/	^723^
			HH1R	Antagonist	/	
			ADRA1	Antagonist	/	
			ADRA2	Antagonist	/	
Promazine		Schizophrenia	DRD2	Antagonist	/	^724–726^
			5-HT-2A	Antagonist	/	
			5-HT-2C	Antagonist	15.87	
			ADRA1A	Antagonist	/	
			CHRM1	Antagonist	/	
			HH1R	Antagonist	2	
Risperidone		Schizophrenia; bipolar disorder	5-HT-2A	Antagonist	/	^727^
			DRD2	Antagonist	/	
			ADRA1B	Antagonist	/	
			ADRA2B	Antagonist	/	
			ADRA1A	Antagonist	/	
			ADRA2C	Antagonist	/	
			HH1R	Antagonist	/	
			5-HT-2C	Antagonist	/	
			5-HT-1D	Antagonist	/	
			5-HT-1A	Antagonist	/	
			5-HT-7	Antagonist	/	
			DRD1	Antagonist	/	
			DRD2	Antagonist	/	
Samidorphan		Schizophrenia	MOR-1	Antagonist	/	/
			KOR-1	Partial agonist	/	
			DOR-1	Partial agonist	/	
Sertindole		Schizophrenia	DRD2	Antagonist	0.45	^728–730^
			5-HT-2A	Antagonist	0.14	
			5-HT-2C	Antagonist	0.2	
			5-HT-6	Antagonist	5	
			ADRA1A	Antagonist	/	
			ADRA1B	Antagonist	/	
			ADRA1D	Antagonist	/	
Sulpiride		Schizophrenia	DRD2	Antagonist	51	^731–733^
			DRD3	Antagonist	8	
			DRD4	Antagonist	/	
Thioproperazine		Schizophrenia	DRD2	Antagonist	/	^622,734^
			ADRA1A	Antagonist	/	
			ADRA1B	Antagonist	/	
			DRD1	Antagonist	/	
Thiothixene		Schizophrenia	DRD2	Antagonist	/	^734^
			DRD1	Antagonist	/	
			5-HT-2A	Antagonist	/	
Trifluoperazine		Schizophrenia	DRD2	Antagonist	/	^622,628,630^
			ADRA1A	Antagonist	/	
Ziprasidone		Schizophrenia	DRD2	Antagonist	2.8	^608,611,619,623,664,694,700,735–738^
			DRD1	Antagonist	9.5	
			DRD5	Antagonist	/	
			DRD3	Antagonist	7.2	
			DRD4	Antagonist	32	
			5-HT-2A	Antagonist	0.08	
			5-HT-1A	Antagonist	1.9	
			5-HT-1B	Antagonist	0.99	
			5-HT-1D	Antagonist	2.4	
			5-HT-1E	Antagonist	360	
			5-HT-2C	Antagonist	0.55	
			5-HT-3	Antagonist		
			5-HT-6	Antagonist	60.9	
			5-HT-7	Antagonist	/	
			5-HT-5A	Antagonist	/	
			HH1R	Antagonist	4.6	
			ADRA1A	Antagonist	/	
			ADRA1B	Antagonist	/	
			ADRA2A	Antagonist	154	
			ADRA2B	Antagonist	48	
			ADRA2C	Antagonist	59	
			CHRM1	Antagonist	300	
			CHRM2	Antagonist	2440	
			CHRM3	Antagonist	1300	
			CHRM4	Antagonist	1600	
			CHRM5	Antagonist	/	
Zuclopenthixol		Schizophrenia	DRD2	Antagonist	/	^739,740^
			DRD1	Antagonist	/	
			DRD5	Antagonist	/	
			ADRA1A	Antagonist	/	
			ADRA2A	Antagonist	/	
			5-HT-2A	Antagonist	/	
			HH1R	Antagonist	/	
Amisulpride		Schizophrenia	5-HT-7	Antagonist	/	^664,741–748^
			5-HT-2A	Antagonist	8304	
			DRD2	Antagonist	/	
			DRD3	Antagonist	/	
			MOR-1	Agonist	/	
			DOR-1	Agonist	/	
			KOR-1	Agonist	/	
Amoxapine		Depression	DRD2	Antagonist	/	^681,749–752^
			DRD1	Antagonist	/	
			ADRA2	Antagonist	/	
			ADRA1	Antagonist	/	
			5-HT-2A	Antagonist	1.77	
			5-HT-2C	Antagonist	/	
			5-HT-6	Antagonist	50	
			5-HT-7	Antagonist	500	
			DRD3	Antagonist	/	
			DRD4	Antagonist	34	
			HH1R	Antagonist	/	
			CHRM	Antagonist	/	
			5-HT-2B	Antagonist	/	
			5-HT-3A	Antagonist	/	
			5-HT-1A	Antagonist	221	
			5-HT-1B	Antagonist	/	
			HH4R	Binder	5012	
Amphetamine		Depression; attention deficit hyperactivity disorder	TAAR1	Agonist	/	^462,753–760^
			ADRA2	Agonist	/	
			ADRA1	Agonist	/	
			ADRB	Agonist	/	
			DRD2	Binder	/	
Buspirone		Depression; anxiety disorders	5-HT-1A	Partial agonist	6.6	^406,629,761–765^
			DRD2	Antagonist	13	
			DRD3	Antagonist	/	
			DRD4	Antagonist	/	
			ADRA1	Partial agonist	/	
Citalopram		Depression; anxiety disorder	HH1R	Binder	/	^409^
			5-HT	Antagonist	/	
Escitalopram		Depression; anxiety disorders	CHRM1	/	/	^766–769^
			HH1R	Inhibitor	/	
			5-HT-1A	Inhibitor	/	
			5-HT-2A	Inhibitor	/	
			ADRA1	Inhibitor	/	
			5-HT-2C	Inhibitor	/	
			ADRA2	Inhibitor	/	
			DRD2	Inhibitor	/	
Paroxetine		Depression; anxiety disorders	5-HT-2A	Agonist	>10000	^428,770–772^
			ADRA1	Binder	/	
			ADRA2	Binder	/	
			ADRB	Inhibitor	/	
			DRD2	Other/unknown	/	
			HH1R	Inhibitor	/	
			5-HT	/	/	
			CHRM	/	/	
Hydroxyzine		Anxiety disorders	HH1R	Inverse agonist	/	^773,774^
Clomipramine		Depression; schizophrenia; Tourette’s disorder	5-HT-2A	Antagonist	35.5	^775,776^
			5-HT-2B	Antagonist	/	
			5-HT-2C	Antagonist	64.6	
Desipramine		Depression; attention deficit hyperactivity disorder; anxiety disorders	5-HT-2A	Antagonist	160	^628,630,777–780^
			ADRB2	Antagonist	/	
			ADRB1	Other/unknown	/	
			HH1R	Antagonist	60	
			ADRA1	Antagonist	/	
			CHRM1	Antagonist	110	
			CHRM2	Antagonist	66	
			CHRM3	Antagonist	210	
			CHRM4	Antagonist	160	
			CHRM5	Antagonist	/	
			5-HT-1A	Binder	6400	
			5-HT-2C	Binder	350	
			DRD2	Binder	/	
			ADRA2	Binder	/	
Dosulepin		Depression; anxiety disorders	5-HT-1A	Antagonist	/	^781^
			5-HT-2A	Antagonist	/	
			HH1R	Antagonist	/	
			CHRM1	Antagonist	/	
			CHRM2	Antagonist	/	
			CHRM3	Antagonist	/	
			CHRM4	Antagonist	/	
			CHRM5	Antagonist	/	
			ADRA2	Antagonist	/	
			ADRA1	Antagonist	/	
Doxepin		Depression; anxiety disorders	HH1R	Antagonist	0.09	^233,383,782,783^
			HH1R	Antagonist	/	
			5-HT-2A	Antagonist	/	
			5-HT-2B	Antagonist	/	
			5-HT-2C	Antagonist	27	
			CHRM1	Antagonist	38	
			CHRM2	Antagonist	23	
			CHRM3	Antagonist	52	
			CHRM4	Antagonist	82	
			CHRM5	Antagonist	/	
			ADRA1A	Antagonist	/	
			ADRA1B	Antagonist	/	
			ADRA1D	Antagonist	/	
			5-HT-1A	Antagonist	276	
			5-HT-6	Binder	105	
			HH4R	Binder	105.9	
Ephedrine		Depression	ADRA1A	Agonist	/	^784–786^
			ADRB1	Agonist	/	
			ADRB2	Agonist	/	
Fluoxetine		Depression	5-HT-2C	Antagonist	112.2	^463,464^
Flupentixol		Depression	DRD2	Antagonist	/	^628,630,787–789^
			DRD1	Antagonist	3	
			5-HT-2A	Antagonist	/	
			ADRA1A	Antagonist	/	
			DRD3	Antagonist	/	
			DRD4	Antagonist	/	
			5-HT-2C	Antagonist	/	
			CHRM1	Antagonist	/	
Imipramine		Depression; attention deficit hyperactivity disorder	5-HT-2A	Antagonist	94	^671,680,790,791^
			HH1R	Antagonist	16	
			ADRA1A	Antagonist	/	
			ADRA1D	Antagonist	/	
			CHRM1	Antagonist	42	
			CHRM2	Antagonist	0.13	
			CHRM3	Antagonist	60	
			CHRM4	Antagonist	112	
			CHRM5	Antagonist	/	
			5-HT-2C	Antagonist	150	
			ADRA1B	Antagonist	/	
			5-HT-7	Antagonist	/	
			DRD1	Binder	/	
			DRD2	Antagonist	726	
			5-HT-1A	Activator	5800	
			5-HT-6	Binder	/	
Maprotiline		Depression; anxiety disorders	HH1R	Antagonist	0.79	^680^
			CHRM1	Antagonist	/	
			CHRM2	Antagonist	/	
			CHRM3	Antagonist	/	
			CHRM4	Antagonist	/	
			CHRM5	Antagonist	/	
			ADRA1	Antagonist	/	
			5-HT-2A	Binder	/	
			5-HT-2C	Binder	/	
			5-HT-7	Antagonist	/	
			DRD2	Binder	/	
			ADRA2	Antagonist	/	
Mianserin		Depression	ADRA2A	Antagonist	4.8	^413,680,792–794^
			5-HT-2A	Antagonist	1.58	
			HH1R	Antagonist	0.36	
			HH4R	Binder	750	
			5-HT-1A	Blocker	398.1	
			5-HT-2C	Antagonist	0.63	
			ADRA2C	Antagonist	3.8	
			5-HT-2B	Binder	/	
			5-HT-1F	Binder	12.58	
			ADRA2B	Antagonist	27	
			DRD3	Binder	2841	
			KOR-1	Agonist	/	
			5-HT-7	Antagonist	56	
			DRD2	Antagonist	2197	
			5-HT-6	Binder	55	
			ADRA1	Antagonist	/	
			DRD1	Binder	/	
Mirtazapine		Depression	5-HT-2A	Antagonist	69	^408,413,414,680,795–799^
			ADRA2A	Antagonist	20	
			ADRA1	Antagonist	/	
			5-HT-3	Antagonist	/	
			5-HT-2C	Antagonist	39	
			KOR-1	Agonist	/	
			HH1R	Antagonist	1.6	
Notriptyline		Depression	5-HT-2A	Antagonist	/	^420,421^
			5-HT-1A	Antagonist	294	
			HH1R	Antagonist	6.3	
			ADRA1A	Antagonist	/	
			ADRA1D	Antagonist	/	
			5-HT-2C	Antagonist	41	
			ADRA1B	Antagonist	/	
			ADRA2	Antagonist	/	
			ADRB	Antagonist	/	
			DRD2	Antagonist	/	
			5-HT-1C	Antagonist	/	
			CHRM	Antagonist	/	
Nefazodone		Depression	5-HT-2A	Antagonist	5.8	^410,411,628,795^
			5-HT-2C	Antagonist	26	
			5-HT-1A	Antagonist	80	
			ADRA1B	Other/unknown	/	
			ADRA2A	Antagonist	84	
			ADRA1A	Antagonist	/	
Voltioxetine		Depression	5-HT-3A	Antagonist	/	^415^
			5-HT-7	Antagonist	/	
			5-HT-1B	Partial agonist	/	
			5-HT-1A	Agonist	/	
			ADRB1	Ligand	/	
Propranolol		Anxiety disorders	ADRB1	Antagonist	0.02	^432^
			ADRB2	Antagonist	/	
			ADRB2	Antagonist	186	
			5-HT-1A	Other	55	
			5-HT-1B	Other	56.23	
Perphenazine		Depression; anxiety disorders; schizophrenia	DRD2	Antagonist	/	^629,800,801^
			DRD1	Antagonist	/	
Pindolol		Depression	ADRB1	Partial agonist	0.52	^405,412,629,802,803^
			ADRB2	Partial agonist	/	
			ADRB3	Agonist	44.1	
			5-HT-1A	Antagonist; Inhibitor; Ligand	22.4	
			5-HT-1B	Ligand; Other/unknown	2600	
Pipradrol		Depression	DRD1	Agonist	/	
Trimipramine		Depression	5-HT-2A	Agonist	/	^629^
			5-HT-1A	Antagonist	/	
			ADRA1A	Antagonist	/	
			ADRA1B	Antagonist	/	
			DRD2	Antagonist	/	
			ADRA2B	Other/unknown	/	
			HH1R	Antagonist	1.4	
			5-HT-2C	Antagonist	/	
			5-HT-3A	Binder	/	
			5-HT-1D	Binder	/	
			ADRA2A	Antagonist	/	
			DRD1	Binder	/	
			ADRB	Binder	/	
			CHRM	Binder	/	
			5-HT-1C	Binder		
Pimozide		Tourette’s disorder	DRD2	Antagonist	11.7	^453^
			DRD3	Antagonist	/	
Atomoxetine		Attention deficit hyperactivity disorder	KOR-1	Partial agonist	/	^460^
Bupropion		Attention deficit hyperactivity disorder; depression	5-HT-3A	Negative modulator	/	^418^
Clonidine		Attention deficit hyperactivity disorder; Tourette’s disorder	ADRA2B	Agonist	31.62	^438,804^
			ADRA2C	Agonist	9.33	
			ADRA2A	Agonist	3.8	
			ADRA1A	Agonist	/	
			ADRA1B	Agonist	316.22	
			ADRA1D	Agonist	125.89	
Dextroamphetamine		Attention deficit hyperactivity disorder	TARR1	Agonist	/	^755,760,805^
			ADRA1B	Antagonist	/	
			ADRA1	Inhibitor; Inducer	/	
			ADRA2	Inhibitor; Inducer	/	
Guanfacine		Attention deficit hyperactivity disorder; Tourette’s disorder	ADRA2A	Agonist	50.3	^451^
			ADRA2B	Binder	1020	
Lisdexamfetamine		Attention deficit hyperactivity disorder	TARR1	Agonist	/	^806,807^
Metamfetamine		Attention deficit hyperactivity disorder	TARR1	Agonist	/	^760,807^
			ADRA2A	Agonist	/	
			ADRA2B	Agonist	/	
			ADRA2C	Agonist	/	
Methylphenidate		Attention deficit hyperactivity disorder	5-HT-3A	/	/	^459^
Serdexmethylphenidate		Attention deficit hyperactivity disorder	5-HT-1A	Agonist	/	^458^

*5-HT* 5-hydroxytryptamine receptor, *5-HT-6* 5-hydroxytryptamine receptor 6, *5-HT-1A* 5-hydroxytryptamine receptor 1A, *5-HT-1B* 5-hydroxytryptamine receptor 1B, *5-HT-1C* 5-hydroxytryptamine receptor 1C, *5-HT-1D* 5-hydroxytryptamine receptor 1D, *5-HT-1E* 5-hydroxytryptamine receptor 1E, *5-HT-1F* 5-hydroxytryptamine receptor 1F, *5-HT-2A* 5-hydroxytryptamine receptor 2A, *5-HT-2B* 5-hydroxytryptamine receptor 2B, *5-HT-2C* 5-hydroxytryptamine receptor 2C, *5-HT-3* 5-hydroxytryptamine receptor 3A, *5-HT-3A* 5-hydroxytryptamine receptor 3A, *5-HT-5A* 5-hydroxytryptamine receptor 5A, *5-HT-6* 5-hydroxytryptamine receptor 6, *5-HT-7* 5-hydroxytryptamine receptor 7, *ACM1* muscarinic acetylcholine receptor M1, *ACM2* muscarinic acetylcholine receptor M2, *ACM3* muscarinic acetylcholine receptor M3, *ACM4* muscarinic acetylcholine receptor M4, *ACM5* muscarinic acetylcholine receptor M5, *ADORA1* adenosine receptor A1, *ADORA2A* adenosine receptor A2a, *ADORA2B* adenosine receptor A2b, *ADRA1* alpha-1 adrenergic receptor, *ADRA1A* alpha-1A adrenergic receptor, *ADRA1B* alpha-1B adrenergic receptor, *ADRA1D* alpha-1D adrenergic receptor, *ADRA2* alpha-2 adrenergic receptor, *ADRA2A* alpha-2A adrenergic receptor, *ADRA2B* alpha-2B adrenergic receptor, *ADRA2C* alpha-2C adrenergic receptor, *ADRB* beta adrenergic receptor, *ADRB2* beta-2 adrenergic receptor, *ADRB3* beta-3 adrenergic receptor, *ADRB1 (gene name)* beta-1 adrenergic receptor, *CB-2* cannabinoid receptor 2, *CB-R or CB1* cannabinoid receptor 1, *CHRM* cholinergic receptor muscarinic, *CHRM1* muscarinic acetylcholine receptor M1, *CHRM2* muscarinic acetylcholine receptor M2, *CHRM3* muscarinic acetylcholine receptor M3, *CHRM4* muscarinic acetylcholine receptor M4, *CHRM5* muscarinic acetylcholine receptor M5, *CXC-R4* C-X-C chemokine receptor type 4, *DOR-1* delta-type opioid receptor, *DRD1* D(1A) dopamine receptor, *DRD2* D(2) dopamine receptor, *DRD3* D(3) dopamine receptor, *DRD4* D(4) dopamine receptor, *DRD5* D(5) dopamine receptor, *GABBR1* gamma-aminobutyric acid type B receptor subunit 1, *GABBR2* gamma-aminobutyric acid type B receptor subunit 2, *GPR18* N-arachidonyl glycine receptor, *GPR12* G-protein coupled receptor 12, *GPR55* G-protein coupled receptor 55, *HH1R* histamine H1 receptor, *HH2R* histamine H2 receptor, *HH3R* histamine H3 receptor, *HH4R* histamine H4 receptor, *KOR-1* kappa-type opioid receptor, *MCHR1* melanin-concentrating hormone receptor 1, *MOR-1* mu-type opioid receptor, *S1PR1* sphingosine 1-phosphate receptor 1, *S1PR3* sphingosine 1-phosphate receptor 3, *S1PR4* sphingosine 1-phosphate receptor 4, *S1PR5* sphingosine 1-phosphate receptor 5, *TAAR1* trace amine-associated receptor 1

Overview of the approved drugs targeting GPCR for the treatment of neuropsychiatric disorders. The approved drugs and their affiliated items including structure, indication, GPCR targets, mechanism, binding affinity (*K*_i_) and related references were collected from the DrugBank database (Accessed May 2022).

**Table 3:** Candidate drugs under development

Drug	Structure	Indication	Phase status	NCT	Targets (protein short names)	Mechanism	*K*_i_ (nM)	Reference
Resveratrol		Alzheimer’s disease; schizophrenia; Parkinson’s disease; depression	1; 2; 2; 4	NCT01504854; NCT02062190; NCT03384329; NCT03095105; NCT03093389; NCT03094156; NCT03097211	Mel-1A-R	/	/	^808^
					Mel-1B-R	/	/	
SGS-742		Alzheimer’s disease; schizophrenia; attention deficit hyperactivity disorder	2	NCT00093951	GABBR1	/	/	/
					GABBR2	/	/	
SUVN-502		Alzheimer’s disease	2	NCT02580305	5-HT-6	/	/	/
Nabilone		Alzheimer’s disease	3	NCT02351882	CB-R	Agonist	/	^349^
					CB-2	Agonist	/	
Caffeine		Alzheimer’s disease	3	NCT04570085	adenosine receptors	/	/	^350^
					5-HT-1	Regulator		
Velusetrag		Alzheimer’s disease	1	NCT01467726	5-HT-4	/	/	/
Brexpiprazole		Alzheimer’s disease	3	NCT03620981	DRD2	Partial Agonist	/	^354^
Prazosin		Alzheimer’s disease	3	NCT03710642	ADRA1A	Antagonist	/	^809^
					CB-2	/	/	
					GPR12	Inverse agonist	/	
					GPR18	/	/	
					GPR55	/	/	
					5-HT-1A	/	/	
					5-HT-2A	/	/	
					DOR-1	/	/	
					MOR-1	/	/	
Sarizotan		Parkinson’s disease	2; 3	NCT00009048; NCT00314288; NCT00105508; NCT00105521	DRD2	Partial agonist	/	/
					DRD3	Ligand	/	
					5-HT-1A	/	/	
Melperone		Parkinson’s disease; schizophrenia; anxiety disorders; depression	2; 3	NCT02374567; NCT00125138;	DRD2	Antagonist	/	/
Pardoprunox		Parkinson’s disease	3	NCT00407095; NCT00406588; NCT00335166; NCT00335374; NCT00332917; NCT00269516	DRD2	/	/	/
					DRD3	/	/	
					DRD4	/	/	
					5-HT-1A	/	/	
Piribedil		Parkinson’s disease	3	NCT01007864	DRD2	/	/	^810^
					DRD3	/	/	
Centanafadine		Attention deficit hyperactivity disorder	3	NCT03605849; NCT03605680; NCT03605836; NCT05257265; NCT05279313; NCT05428033	/	/	/	
Raclopride		Parkinson’s disease; depression	1; 4	NCT00832221; NCT05282277	DRD2	Antagonist	/	^811^
Dipraglurant		Parkinson’s disease	2; 2/3	NCT01336088; NCT05116813; NCT04857359	MGLUR5	/	/	/
Arbaclofen Placarbil		Multiple sclerosis	3	NCT01359566	GABBR1	Agonist	/	^812^
					GABBR2	Agonist	/	
Plozalizumab	Biotech	Multiple sclerosis	2	NCT01199640	CMKBR2	/	/	/
Nabiximols		Multiple sclerosis	3; 4	NCT01964547; NCT00678795; NCT00681538; NCT00702468; NCT00711646	GPR12	Inverse agonist	/	^658^
					CB-R	/	/	
					CB-2	/	/	
					GPR55	/	/	
					5-HT-1A	/	/	
					5-HT-2A	/	/	
					DOR-1	/	/	
					MOR-1	/	/	
Ceralifimod		Multiple sclerosis	2	NCT01226745	S1PR1	Modulator	/	^813^
Tiapride		Huntington’s disease; schizophrenia; depression; anxiety disorders	1; 3	NCT00632645; NCT02374567	DRD2	Blocker	/	^814^
					DRD3	Blocker	/	
					5-HT	Antagonist	/	
					ADRA1	Antagonist	/	
					ADRA2	Antagonist	/	
LY2140023	Not Available	Schizophrenia	1; 2; 2/3; 3	NCT01307800; NCT01328093; NCT01487083; NCT01452919; NCT01129674; NCT01125358; NCT01052103; NCT00149292; NCT00520923; NCT00845026; NCT01086748; NCT01606436; NCT01354353	MGLUR2	/	/	/
					MGLUR3	/	/	
BL-1020	Not available	Schizophrenia	2; 2/3	NCT00480571; NCT00722176	DRD2	/	/	/
					5-HT-2A	/	/	
Norclozapine		Schizophrenia	1; 2	NCT00628420; NCT00490516	CHRM1	/	/	/
					DRD2	/	/	
					DRD3	/	/	
Talnetant		Schizophrenia	2	NCT00049946; NCT00103727; NCT00300963; NCT00101985	NK3R	Antagonist	1	^815^
Blonanserin		Schizophrenia	4; 3	NCT01516424; NCT03784222	DRD2	Antagonist	/	^816^
					DRD3	Antagonist	/	
					5-HT-2A	Antagonist	/	
Pipamperone		Schizophrenia; depression; anxiety disorders	2; 3	NCT00672659; NCT01450514; NCT02374567; NCT01312922	DRD2	Antagonist	/	/
					5-HT-2A	Agonist	/	
					ADRA1	Antagonist	/	
					DRD4	Antagonist	/	
					DRD1	Antagonist	/	
					DRD3	/	/	
					5-HT-2B	/	/	
					ADRA2A	Antagonist	/	
Pavinetant		Schizophrenia	2	NCT00686998	NK3R	Antagonist	/	^817^
Tetrahydrocannabivarin		Schizophrenia	2	NCT01491490	CB-R	Antagonist	/	^818^
					GPR55	Partial agonist	/	
					5-HT-1A	Agonist	/	
					CB-2	Partial agonist	/	
JNJ-37822681		Schizophrenia	2	NCT00728195; NCT01812642	DRD2	Antagonist	/	^819^
SEP-363856		Schizophrenia; Parkinson’s disease	1; 2; 2/3; 3	NCT04865835; NCT03370640; NCT04325737; NCT04369391; NCT01940159; NCT01972711; NCT01994473; NCT04038957; NCT02970929; NCT02969382; NCT04825860; NCT05359081; NCT04092686; NCT04072354; NCT04109950; NCT02969369	TAAR1	Agonist	/	^820^
					5-HT-1A	Agonist	/	
Dimethyltryptamine		Depression	1; 1/2	NCT04711915; NCT04698603	5-HT-6	/	68	^821,822^
					5-HT-2A	/	65	
Serotonin		Depression; bipolar disorder; anxiety disorders	2; 3; 2/3; 4	NCT02137369; NCT01324700; NCT01811147; NCT00183274; NCT00157547; NCT02356107’ NCT01155661; NCT00361218	5-HT-2A	/	/	^823^
					5-HT-3A	/	/	
					5-HT-3B	/	/	
5-methoxy-N,N-dimethyltryptamine		Depression	1/2	NCT04698603	5-HT-1A	Agonist	/	^824^
					5-HT-2A	Agonist	/	
Tianeptine		Bipolar disorder; depression	3; 4	NCT00879372; NCT01309776; NCT04249596	MOR-1	Agonist	/	^440,441^
					5-HT-1A	Inhibitor	/	
					DRD3	Agonist	/	
Vofopitant		Bipolar disorder	1	NCT00907985	SPR	/	/	^825^
Naluzotan		Anxiety disorders; depression	3; 2	NCT00248183; NCT00448292	5-HT-1A	Agonist	/	^435^
Ansofaxine		Depression	3	NCT04853407	5-HT	/	/	/
Roluperidone		Schizophrenia	3	NCT03397134	5-HT-2A	/	/	^402^
Eltoprazine		Schizophrenia; Parkinson’s disease (PD)	2	NCT01266174; NCT02439125	5-HT-1A	/	/	^401^
					5-HT-2B	/	/	
Zicronapine		Schizophrenia	3	NCT01295372	5-HT-2A	/	/	^400^
					5-HT-2C	/	/	
					DRD1	/	/	
					DRD2	/	/	
Brilaroxazine		Schizophrenia	2; 3	NCT01490086; NCT05184335	5-HT-7	/	/	^399^
					5-HT-2A	/	/	
					5-HT-1A	/	/	
					DRD2	/	/	
					DRD3	/	/	
					DRD4	/	/	
					5-HT-6	/	/	

*5-HT* 5-hydroxytryptamine receptor, *5-HT-1* 5-hydroxytryptamine receptor 1, *5-HT-6* 5-hydroxytryptamine receptor 6, *5-HT-1A* 5-hydroxytryptamine receptor 1A, *5-HT-1B* 5-hydroxytryptamine receptor 1B, *5-HT-1C* 5-hydroxytryptamine receptor 1C, *5-HT-1D* 5-hydroxytryptamine receptor 1D, *5-HT-1E* 5-hydroxytryptamine receptor 1E, *5-HT-1F* 5-hydroxytryptamine receptor 1F, *5-HT-2A* 5-hydroxytryptamine receptor 2A, *5-HT-2B* 5-hydroxytryptamine receptor 2B, *5-HT-2C* 5-hydroxytryptamine receptor 2C, *5-HT-3* 5-hydroxytryptamine receptor 3A, *5-HT-3A* 6-hydroxytryptamine receptor 3A, *5-HT-3B* 5-hydroxytryptamine receptor 3B, *5-HT-4* 5-hydroxytryptamine receptor 4, *5-HT-7* 5-hydroxytryptamine receptor 7, *ADRA1* alpha-1 adrenergic receptor, *ADRA1A* alpha-1A adrenergic receptor, *ADRA1B* alpha-1B adrenergic receptor, *ADRA1D* alpha-1D adrenergic receptor, *ADRA2* alpha-2 adrenergic receptor, *ADRA2A* alpha-2A adrenergic receptor, *ADRA2B* alpha-2B adrenergic receptor, *ADRA2C* alpha-2C adrenergic receptor, *ADRB2* beta-2 adrenergic receptor, *ADRB3* beta-3 adrenergic receptor, *ADRB1* beta-1 adrenergic receptor, *CB-2* cannabinoid receptor 2, *CB-R or CB1* cannabinoid receptor 1, *CHRM* cholinergic receptor muscarinic, *CHRM1* muscarinic acetylcholine receptor M1, *CHRM2* muscarinic acetylcholine receptor M2, *CHRM3* muscarinic acetylcholine receptor M3, *CHRM4* muscarinic acetylcholine receptor M4, *CHRM5* muscarinic acetylcholine receptor M5, *DOR-1* delta-type opioid receptor, *DRD1* D(1A) dopamine receptor, *DRD2* D(2) dopamine receptor, *DRD3* D(3) dopamine receptor, *DRD4* D(4) dopamine receptor, *DRD5* D(5) dopamine receptor, *GABBR1* gamma-aminobutyric acid type B receptor subunit 1, *GABBR2* gamma-aminobutyric acid type B receptor subunit 2, *GPR18* N-arachidonyl glycine receptor, *GPR12* G-protein coupled receptor 12, *GPR55* G-protein coupled receptor 55, *HH1R* histamine H1 receptor, *HH2R* histamine H2 receptor, *HH3R* histamine H3 receptor, *HH4R* histamine H4 receptor, *KOR-1* kappa-type opioid receptor, *MCHR1* melanin-concentrating hormone receptor 1, *MOR-1* mu-type opioid receptor, *SPR* neurokinin 1 receptor, *S1PR1* sphingosine 1-phosphate receptor 1, *TAAR1* trace amine-associated receptor 1, *Mel-1A-R* melatonin receptor type 1A, *Mel-1B-R* melatonin receptor type 1B, *MGLUR5* metabotropic glutamate receptor 5, *MGLUR2* metabotropic glutamate receptor 2, *MGLUR3* metabotropic glutamate receptor 3, *CMKBR2* C-C chemokine receptor type 2, *NK3R* neuromedin-K receptor

Overview of the clinical stage drugs targeting GPCR for the treatment of neuropsychiatric disorders. The clinical stage compounds and their affiliated items including structure, indication, phase status, NCT number, GPCR targets, mechanism, binding affinity (*K*_i_) and related references were collected from the DrugBank database (Accessed May 2022).

### Neurodegenerative diseases

#### Alzheimer’s disease

Alzheimer’s disease (AD) is a progressive neurodegenerative disease. AD patients present with cognitive deficits, memory loss, and personality and behaviour changes. Currently, there is no curative treatment for AD. Reducing patients’ symptoms and delaying the disease’s progression is the primary objective of treatment. α1-adrenergic receptor, dopamine receptor, muscarinic acetylcholine receptor M3, histamine H1 receptor, and serotonin receptors are the primary therapeutic targets. Medication to control mental symptoms is another important objective, as patients manifest neuropsychiatric symptoms frequently.

Developing new drugs for AD is challenging, with high failure rates and long development periods. Several trials attempt to explore the use of GPCR agonism in AD treatment. SUVN-502 is in the Phase II trial (NCT02580305) to evaluate its safety and efficacy in moderate AD treatment.^346^ SUVN-502 is an orally active 5-HT6 receptor antagonist exhibiting effects by modulating cholinergic and glutamatergic neurotransmission.^347^ ∆9-tetrahydrocannabinol (THC) analog Nabilone (agonist targeting CB1/2 receptor) is under phase III investigation (NCT02351882) for its benefit on agitation, hyperactive behavioural symptoms of AD.^348,349^ Caffeine, the antagonist of adenosine receptor antagonist, could modify brain dysfunctions in various neurodegenerative diseases including AD, Parkinson’s disease, Huntington’s disease. The efficacy of caffeine on cognitive decline in AD dementia is undergoing examination in phase III clinical trial (NCT04570085).^350^

Guanfacine is an α2a-adrenergic agonist.^351^ Guanfacine could increase brain noradrenaline levels. Dual actions of Guanfacine on noradrenergic transmission and thalamocortical glutamatergic transmission have been reported.^352^ Guanfacine is a drug for treating children’s attention deficit/hyperactivity disorder (ADHD). The efficacy for improving cognition in AD is evaluated in the phase III trial (NCT03116126). The α1-adrenergic receptor antagonist, Prazosin, is being tested for its effectiveness on agitation in adults with AD in a phase III trial (NCT03710642). Prazosin is a drug for hypertension, benign prostatic hyperplasia, and post-traumatic stress disorder (PTSD) associated nightmares. Prazosin can cross the blood-brain barrier and act on the active α1-adrenoreceptor in the brain.

Brexpiprazole is classified as a novel class of antipsychotic with serotonin-dopamine modulating functions. It is an atypical antipsychotic that function as a partial agonist for serotonin and dopamine receptors. As a partial agonist, Brexpiprazole exerts smaller responses than the native ligands.^353,354^ The use of Brexpiprazole in AD agitation is now in phase III study (NCT03620981).

#### Parkinson’s disease

Parkinson’s disease (PD) is the second most prevalent age-related disorder. Early stage with mild symptoms did not require medication. Dopamine-like agonists, also known as dopamine-replacement therapy, are the primary treatment for symptomatic PD. As degeneration of the substantia nigra leading to striatal dopamine reduction is a leading cause of PD, re-introducing dopamine can improve motor problems dramatically and slow down PD progression.^348,355^

Levodopa is a dopamine precursor. It has long been used in controlling bradykinetic symptoms in PD. Levodopa can cross the blood-brain barrier and is known as a well-tolerated drug for dopamine-replacement therapy.^356^ However, Levodopa could lead to motor and psychiatric side effects.^357^ Amantadine could reduce dyskinesia (involuntary movements) in PD patients receiving Levodopa.^358^ Amantadine is an antiviral medicine with antiparkinsonian effects. Synergistic effects are observed when used in combination with Levodopa.^359,360^ Lisuride functions as a dopamine receptor agonist with 5-HT1A receptor agonist and 5-HT2B receptor antagonist for PD treatment.^361^ Piribedil is a dopamine agonist used with or without Levodopa in a phase III trial to treat idiopathic PD (NCT01519856).^362^ Bromocriptine is a dopamine D2 receptor agonist for early PD treatment. Bromocriptine works by activating post-synaptic dopamine receptors.^363^

Apomorphine is a morphine derivative. It functions as a D2 dopamine agonist for treating hypermobile “off” episodes of advanced PD, a stage in which PD symptoms get worse even with scheduled medication. It also prevents dyskinesia by functioning as a 5-HT1A receptor agonist.^364^ The A2A receptor in the basal ganglia is involved in the motor control of PD.^365^ At present, Istradefylline is the principal adenosine A2A receptor antagonist employed in adult PD patients presenting “off” episodes associated with Levodopa treatment.^141^

Pergolide is a long-acting dopamine receptor agonist approved in 1982 for treating PD. It functions on various GPCRs, including dopamine D2/3 receptor, α1/2-adrenergic receptor, and 5-HT receptors. It is used as adjunct therapy with Levodopa and carbidopa in the symptomatic treatment of PD.^366^ Ropinirole is a non-ergoline dopamine agonist, approved as monotherapy and as an adjunct to Levodopa in the treatment of PD.^367^

Benztropine is used to treat the molecular mechanism of anticholinergics PD.^368^ Benztropine inhibits dopamine uptake and exhibits varied binding affinities for muscarinic acetylcholine M1 and histamine H1 receptors.^369^ Biperiden, another anticholinergic drug launched in 1954, has an antagonistic effect on the muscarinic acetylcholine receptor.^368^

Pramipexole is a non-ergot-derived dopaminergic agonist for PD treatment. Pramipexole treatment enhances DA and 5-HT neurotransmission and increases tonic activation of post-synaptic D2 and 5-HT1A receptors in the forebrain.^370^ Apart from PD, Pramipexole can also be prescribed for psychiatric conditions such as treatment-resistant depression and bipolar disorder.^371^

#### Multiple sclerosis

Multiple sclerosis (MS) results from an immune attack by infiltrating inflammatory leukocytes in the central nervous system, causing hard, mottled pathologic changes and nerve conduction disorders.^372,373^ At present, medication aims to control GPCR-regulated immune cell function as one of the treatment regime for MS. In the database, 6 GPCR-related drugs are recorded. The drugs target multiple GPCRs, including adrenergic receptors, cannabinoid receptors, dopamine receptors, GABA receptors, opioid receptors, orphan GPCRs (GPR12/18/55), S1PR1/5, and chemokine receptors.

Baclofen is a derivative of the neurotransmitter γ-aminobutyric acid (GABA). Baclofen can help relax the stiff muscle (muscle spasticity) experienced by MS patients. Cannabidiol (CBD), one of the active components in cannabis, could improve mobility in MS by reducing depression, fatigue, inflammation, pain, and spasticity (stiff muscle with feelings of pain or tightness) in MS patients.^374^ Modafinil is a partial agonist for brain α1b-adrenoceptor. Pharmacological blockade of α1b-adrenoceptor shows benefit in controlling fatigue syndromes in MS. Modafinil exhibits clinical efficacy in psychiatric conditions, including treatment-resistant depression and attention deficit/hyperactivity disorder.^375^

Ozanimod, Siponimod, and Fingolimod are S1PR agonists that selectively bind to the S1PR1 and S1PR5 subtypes, inhibiting lymphocyte egress from lymph nodes.^376^ Ozanimod demonstrates a favourable safety profile in trials.^377^ Fingolimod may cause undesirable effects because of its interaction with other S1PR subtypes. Compared to Fingolimod, Siponimod has fewer off-target effects.

Ceralifimod is a selective S1PΡ1/5 agonist under investigation in phase II clinical trial NCT01226745 in patients with relapsing-remitting multiple sclerosis (a condition with relapses or exacerbations of old and new symptoms).^267^ Plozalizumab is another potential drug for MS treatment. It is a humanized anti-CCR2 monoclonal antibody targeting white blood cells.^378^ Plozalizumab may regulate inflammatory responses by targeting the CCL2‐CCR2 axis in MS.

#### Huntington’s disease

Huntington’s disease (HD) is a hereditary neurodegenerative disease. Symptoms include movement disorders and cognitive and psychiatric manifestations. Blocking and antagonizing dopamine are effective for HD treatment. Tetrabenazine is a reversible vesicular monoamine transporter 2 (VMAT) inhibitor that inhibits the reuptake of neurotransmitters in presynaptic neurons. VMAT helps to repackage the unbound dopamine taken up by the pre-synaptic terminal. Although it is first designed for schizophrenia treatment, clinical trials demonstrate efficacy in treating hyperkinetic movement disorders.^379^ Tetrabenazine also functions as a D2 post-synaptic receptor blocker at high doses and is used to treat uncontrolled muscle movement in HD.^379^ Haloperidol is a first-generation antipsychotic for schizophrenia and psychotic disorders.^380^ As a dopamine receptor antagonist, Haloperidol is used off-label for managing chorea associated with HD.^381^ For cognitive impairment, no effective targeted therapy is available at the present stage. Tiapride is in phase III for the treatment of HD (NCT00632645). Preclinical pharmacologic and behavioral research suggests that Tiapride is a selective blocker of dopamine D2 and D3 receptors in limbic brain regions.^382^

### Psychiatric disorders

#### Schizophrenia

Schizophrenia is characterized by cognitive deficits and positive and negative symptoms with complex inheritance patterns.^383^ Patients may have positive, negative, cognitive, and general psychopathological disorders. According to the positive and negative syndrome scale (a psychiatric rating system), positive symptoms include delusions, hallucinations, conceptual disorganization, hallucinatory, excitement, grandiosity, suspiciousness, and hostility; Negative symptoms include blunted affect, emotional withdrawal, poor rapport, passive social withdrawal, difficulty in abstract thinking or stereotyped thinking and lack of spontaneity and flow of conversation. Schizophrenia patients could also present general cognitive disorders. Examples include anxiety, guilt feeling, tension, depression, poor attention or impulse control, and active social avoidance.^384^

Schizophrenia treatment is challenging because existing antipsychotics are antidopaminergic drugs that improve only positive symptoms such as agitation and aggression but have limited efficacy for negative and cognitive symptoms.^385^ Globally marketed antipsychotic drugs include typical antipsychotic drugs (mostly specific dopamine D2 receptor antagonists) and atypical antipsychotic drugs (such as dopamine D2 and 5-HT2A dual antagonists and D2/D3 partial agonists).

Aripiprazole, a blockbuster drug for controlling psychiatric symptoms, has high affinities for 5-HT1A, 5-HT2A, D2, and D3 receptors. It is a partial agonist of D2, D3, and 5-HT1A receptors and a 5-HT2A receptor antagonist.^386^ Aripiprazole is also a drug for bipolar disorders.^387^ Brexpiprazole, developed by Otsuka, is considered as the pharmacological successor to Aripiprazole. Brexpiprazole can also be used as an adjunct for major depressive disorder.^388–390^

Cariprazine is a D3/D2 partial agonist with moderate affinity for the 5-HT2A receptor.^391^ FDA approved it in 2016 for treating adult schizophrenia and bipolar disorder.

Lumateperone is an antipsychotic targeting multiple GPCRs. It is a post-synaptic dopamine D2 receptor antagonist, a presynaptic dopamine D2 receptor partial agonist, and a 5-HT2A receptor antagonist.^392^ Lumateperone can be used for positive & negative symptoms and cognitive dysfunction in schizophrenia.^393^ It can also be used in bipolar disorder treatment.^393^

Chlorpromazine blocks dopamine receptors, α-adrenergic receptors, and 5-HT receptors. It can quickly control the state of agitation and gradually eliminate hallucinations and delusions. Thus, it can apply as medication to control combativeness and aggressive behaviour in children.^394^

Risperidone can be used for various mental disorders, including schizophrenia and mood disorders. Risperidone has high affinities for 5-HT receptors and dopamine receptors and mildly inhibits α1-adrenergic receptors and histamine receptors.^395^

Olanzapine is developed based on clozapine with structural modification. It was approved to be marketed by FDA in 1996. Olanzapine not only inhibits dopamine receptors but also binds to serotonin receptors, and its affinity with serotonin receptors is far greater than its affinity with dopamine receptors.

Haloperidol is a widely used antipsychotic for positive symptoms of schizophrenia, Tourette syndrome, and behavioural disorders/hyperactivity in children.^396^ Haloperidol can block dopamine, α-adrenergic, and serotonin receptors. It is highly selective for dopamine receptors.

Spiperone is a potent dopamine D2 receptor antagonist bearing the butyrophenone scaffold. Although it displayed efficacy in treating drug-resistant schizophrenia, it is not yet approved by the FDA.^397^ Zotepine is an atypical antipsychotic drug for treating schizophrenia in Japan. It is a potent dopamine D1/D2 receptor and 5-HT2A receptor antagonist.^398^

Medication for schizophrenia is an active research area. Schizophrenia drugs generally target multiple GPCRs. For instance, Brilaroxazine, an investigational antipsychotic drug developed by Reviva, could stabilize the dopamine-serotonin system by partially activating D2, D3, D4, 5-HT1A, and 5-HT2A receptors. In addition, it antagonizes 5-HT6 and 5-HT7 receptors.^399^ A phase III clinical trial of Brilaroxazine for the safety and efficacy of the treatment of schizophrenia is now under recruitment (NCT05184335).

Zicronapine is a tetracyclic azepine developed by Lundbeck with affinities for 5-HT2A/2 C and D1/2 receptors.^400^ Phase III study of Zicronapine has been completed (NCT01295372).

Eltoprazine is a piperazine derivative that partially activates the 5-HT1A/2B receptor.^401^ It is tested in a phase II trial to investigate the treatment of schizophrenia and cognitive impairment (NCT01266174).

LuAF35700 is an antagonist targeting dopamine receptors, serotonin receptors, and α-adrenergic receptors.^399^ The efficacy and safety of the LuAF35700 have been examined in phase III randomized, double-blind trial (NCT02717195).

Roluperidone is a novel 5-HT2A and σ2 receptor antagonist developed by Minerva Neurosciences.^402^ Phase III studies have shown that Roperidone may treat negative symptoms in schizophrenia patients without causing post-synaptic dopaminergic blockade due to low or no affinity for dopamine and histamine receptors (NCT03397134).

#### Depression

The underlying mechanism of depression is not clear. According to the record in the DrugBank database, a total of 31 antidepressants target GPCRs. Examples include tricyclic antidepressants, bioamine neurotransmitters (serotonin, norepinephrine, and dopamine) reuptake blockers, and 5-HT2A receptor inhibitors.

Imipramine and Desipramine are examples of tricyclic drugs for major depressive disorders, anxiety, and ADHD.^403^ They have high affinities to 5-HT2C and 5-HT2A receptor subtypes. The pharmacological properties of Amitriptyline are similar to Imipramine. Amitriptyline can inhibit 5-HT reuptake with sedative, hypnotic and anticholinergic effects. A combination of Amitriptyline and Imipramine could block serotonin reuptake in the brain’s limbic (emotional) regions.

Currently, monoaminergic alterations involving serotonin receptors are a significant cause of depression.^404^ Selective or non-selective 5-HT reuptake inhibitors are the first-line treatment for depression. Representative drugs include Fluoxetine, Paroxetine, and Citalopram.^405–409^ Fluoxetine, a weak antagonist of 5-HT2C and 5-HT2A receptors, was approved for marketing in 1988 to treat major depressive disorder. Later, Paroxetine was approved in 1992. It is a highly selective reuptake inhibitor of 5-HT in neurons. Citalopram has a similar function in depression treatment. It is also a serotonin reuptake inhibitor. Nefazodone and Trazodone improve mood by antagonizing 5-HT2A/C receptors. They showed affinity to the 5-HT1A receptor.^410,411^ Pindolol can accelerate the effects of selective serotonin reuptake by antagonizing 5-HT1A and β-adrenergic receptors.^405,412^ Meanwhile, Mirtazapine and Mianserin have antagonistic properties on 5-HT2A/2C receptors. They exhibit inhibitory effects on presynaptic A2-adrenergic receptors. Both drugs improve sleep duration.^408,413,414^ Vortioxetine is a multi-mode antidepressant for major depressive disorder treatment in adults. Vortioxetine inhibits serotonin reuptake. It exerts different effects on different members of the 5-HT receptor. On one hand, Vortioxetine is an antagonist for 5-HT1D, 5-HT3, and 5-HT7 receptors. On the other hand, it is a partial agonist for the 5-HT1B receptor.^415–417^ Bupropion and its primary metabolite hydroxybupropion function by blocking 5-HT3A receptor.^418^ Agomelatine is an atypical antidepressant acting as a melatonin receptor (MT1/2) agonist and a 5-HT2C/2B receptor antagonist.^419^

Inhibitors of dopamine (DA) transporters are another class of antidepressants. Nortriptyline can bind directly to the DA transporter to inhibit dopamine uptake. It can be used in treatment-resistant depression.^420–422^ Brexpiprazole is a partial agonist on the 5-HT1A receptor and D2 receptor. Brexpiprazole can also be used in adult patients with schizophrenia.

Ansofaxine is a reuptake inhibitor for 5-HT, norepinephrine, and dopamine which is under clinical development for major depressive disorder (NCT04853407).^423^ 5-methoxy-N, N-dimethyltryptamine (5-MEO-DMT) is a non-selective serotonin receptors agonist for depression (NCT04698603).

#### Anxiety disorders

Anxiety disorders are the most common psychiatric disorders. Anxiety is accompanied by other psychiatric disorders, including major depressive disorders, substance use disorders, and personality disorders.^424^

Partial agonists of the 5-HT1A receptor and selective 5-HT reuptake inhibitors are frequently used in anxiety treatment.^425,426^ Buspirone, the partial agonist for the 5-HT1A receptor, is approved for treating anxiety due to neurosis.^427^ Paroxetine^428^ and Escitalopram, the 5-HT reuptake inhibitors, can relieve anxiety symptoms and prevent recurrence in patients.^409^ Trazodone is used to treat anxiety disorders with depressive symptoms and is suitable for patients with significant psychomotor agitation, anxiety, and insomnia.^429^

Hydroxyzine is the most studied antihistamine for anxiety and the only FDA-approved antihistamine for treating anxiety. It is commonly used for anxiety, panic attacks, and insomnia in inpatients and outpatients.^429,430^

Drug targeting β-adrenoreceptor in the central nervous system can also relieve anxiety.^431^ Propranolol, the selective β1/2-adrenoceptor antagonist (β-blockers), is the first-line pharmacological treatment for anxiety disorders.^432,433^ Doxepin can be used for depression and anxiety. It is an antagonist of the histamine H1 and H2 receptors, 5-HT2A/2C receptors, and the muscarinic acetylcholine receptors (M1–M5).^434^

Naluzotan, the selective 5-HT1A receptor agonist, has been investigated for anxiety disorders and depression treatment (NCT00248183).^435^ Ansofaxine, a reuptake inhibitor of serotonin, norepinephrine, and dopamine, is a new-generation drug for anxiety management. The drug has completed phase III clinical trials in China to treat anxiety and depression (NCT04853407).

#### Bipolar disorder

Bipolar disorder (BD) is characterized by periodic mood disorders. Medication is the primary treatment to improve the psychosocial function and quality of life of patients with BD. Pharmacological management of acute depressive/manic episodes and prevention of recurrence is also essential. Atypical antipsychotics for bipolar disorder exhibit high affinities for multiple serotonergic receptors, including 5-HT1A, 5-HT2A-C, 5-HT6, and 5-HT7 receptors.

Quetiapine was approved by the FDA in 1997 for the symptomatic treatment of schizophrenia and is used as a first-line treatment to control depressive episodes of BD. It exerts therapeutic effects may by antagonizing 5-HT1A, 5-HT2A, D1, D2, and H1 receptors as well as α1/2- adrenergic receptors.^436,437^ Dexmedetomidine is an α2-adrenergic receptor agonist that can be used for the acute treatment of agitation associated with schizophrenia or bipolar I or II disorders.^438^ Risperidone, an atypical antipsychotic drug, is now used as maintenance therapy for patients with bipolar I disorder.^439^

Tianeptine is a novel antidepressant that stimulates serotonin, increases levels of 5-hydroxyindoleacetic acid in brain tissue and plasma, and decreases serotonin-induced behavior.^440,441^ Clinical trials are underway for the adjuvant treatment for BD with Tientidine (NCT00879372). Lumateperone, an antagonist with high binding affinity to the 5-HT2A receptor and moderate affinity to the post-synaptic D2 receptor, is being evaluated for treating BD, depression, and other neuropsychiatric and neurological disorders (NCT03249376, NCT02600507).

#### Tourette’s syndrome

Tourette’s syndrome (TS) is a neurodevelopmental disorder characterized by repetitive behaviours, including motor/phonic tics. TS is commonly coupled with obsessive-compulsive disorder (OCD) and ADHD.^442^ The underlying mechanism of TS remains poorly clarified.^443–445^ Abnormalities in synaptic neurotransmission involved in the cortico-striatal-thalamocortical circuitry are implicated in TS pathogenesis.^446,447^ Dopaminergic signaling in cortico-striatal-thalamocortical pathways might be associated with TS progression.^444,448,449^ α-adrenergic agonists are the first choice in TS treatment.^450^ Examples include Clonidine and Guanfacine.^438,451^ Aripiprazole is a partial agonist of dopamine D2 and 5-HT1A receptors. It can stabilize dopamine receptor and improves TS symptoms.^452^ In contrast, Pimozide exerts a therapeutic effect by inhibiting the dopamine D2 receptor in the central nervous system.^453^

#### Attention deficit hyperactivity disorder

Attention deficit hyperactivity disorder (ADHD) is a common psychiatric disorder affecting school-age children. It is a neurodevelopmental disorder with multifactorial etiological risk factors. ADHD is characterized by hyperactivity, impulsivity, and age-inappropriate symptoms of inattention.^454^ Irregularities in catecholamines circuits in the prefrontal cortex, such as dopamine and norepinephrine, are a leading cause of ADHD.^455,456^ Most ADHD drugs are designed to enhance catecholamine transmission in the prefrontal cortex.^457^

Methylphenidate can significantly reduce hyperactive behavior, increase attention concentration ability, and effectively improve the core symptoms of ADHD, so it is one of the most widely used first-line drugs approved by the FDA. Methylphenidate blocks dopamine D1 and D2 transporters, resulting in increased levels of synaptic dopamine, and also shows activity against serotonergic 5-HT1A receptors.^351,458,459^

Second-line drugs for ADHD include Atomoxetine, Guanfacine, and Clonidine.^351,438,460^ Atoroxetine is a non-stimulant medication that acts as a selective norepinephrine reuptake inhibitor in ADHD.^440,461^ Guanfacine is a phenylacetyl guanidine derivative, which is more selective than Clonidine in activating the α2-adrenergic receptor.^351^ Venlafaxine is a new type of selective serotonin and dopamine reuptake inhibitor. It is a dual-channel antidepressant. Venlafaxine inhibits the reuptake of serotonin by neuron endings at low doses and inhibits the reuptake function of neuron endings at a high dose to enhance attention. Amfetamine (AMF) acts on the cerebral cortex and reticular activation system. AMF stimulates adrenalin receptors and enhances neurotransmitter secretion, such as 5-HT and dopamine.^462^ Fluoxetine is a potent and selective serotonin reuptake inhibitor for ADHD treatment.^463,464^

Edivoxetine is an adrenergic absorption inhibitor. It is now in phase III development for ADHD with hyperactivity (NCT00922636, NCT00965419). Centanafadine is a triple-reuptake inhibitor for dopamine, norepinephrine, and serotonin reuptake. It is currently in phase III clinical trials (NCT03605849, NCT03605680, NCT03605836). SGS-742 has been investigated for ADHD treatment. It acts as a GABA-B receptor antagonist and could enhance the release of glutamate, aspartate, glycine, and somatostatin.

## Example of emerging GPCR targets

Most of the GPCRs targeted by approved drugs for neuropsychiatric diseases belong to class A and C GPCRs. With the advance of biotechnology and increase in understanding of GPCR functions, new candidates are discovered in other GPCR families, including class A (orphan), class B1 (secretin), class B2 (adhesion), class C (calcium-sensing receptor), and class F.

### Class A (orphan GPCR)

Orphan GPCRs are receptors whose cognate ligands are not discovered or validated in cellular/ animal models. Deorphanization with reverse pharmacology is currently an active area in GPCR research.

#### GPR17

GPR17 is activated by two different endogenous ligands: uracil nucleotides and cysteinyl-leukotrienes.^465^ Uracil nucleotides trigger astrocytic migration by upregulating membrane integrins.^466^ Cysteinyl-leukotrienes are lipid mediators secreted by inflammatory cells and nervous tissues.^467^ Cysteinyl‐leukotrienes can stimulate astrocyte proliferation via autocrine signaling.^468^ GPR17 is a sensor of local damage to the myelin sheath. GPR17 downregulation promotes the development of mature oligodendrocytes from myelin-producing oligodendrocyte precursors.^469^ GPR17 is involved in reconstructing and repairing demyelinating plaques formed by ongoing inflammatory processes.^470^ In a mouse model of multiple sclerosis, targeting GPR17 can delay the onset of autoimmune encephalomyelitis.^471^

#### GPR26

GPR26 is a brain-specific GPCR. GPR26 has high sequence homology with purinergic P2Y receptor and serotonin 5-HT5A receptor.^472,473^ GPR26 regulates emotion in animal models. GPR26 knockout mice exhibits anxiety- and depressive-like behaviors.^474^ Colocalization of GPR26 and neuronal nuclear inclusions is observed in brain tissues suggesting a potential link between GPR26 and neurodegenerative diseases.^473^

#### GPR37 and GPR37L1

GPR37 can be found in pre-myelinating/myelinating oligodendrocytes, dopaminergic neurons, and hippocampal neurons.^475^ GPR37 shares high sequence homology with peptide-activated GPCRs such as endothelin receptor B (ETB).^475^ In Parkinson’s disease, GPR37 acts as an adenosine A2A receptor inhibitor via receptor oligomerization;^476^ GPR37L1, in contrast, is found mainly in astrocytes and oligodendrocyte progenitor cells.^475^ GPR37L1 is involved in the adaptive myelination of oligodendrocytes which is critical for neural plasticity, learning, and memory in adults.^477^

#### GPR39

Zinc regulates behavior, cognition, and ability to learn.^478^ Dysregulation in zinc homeostasis is associated with progressive dementia and cognitive impairment. Zinc deficiency gives rise to various neuropsychiatric disorders, including epilepsy, seizures, and depression.^479,480^ Extracellular zinc can activate zinc-sensing receptor GPR39.^481,482^ Zinc stimulates GPR39-mediated signal transduction and induces calcium mobilization in HEK293 cells.^483^ Zinc-activated GPR39 increases expression of K^+^/Cl^−^ cotransporter 2 (KCC2), the Cl^-^ outward transporter in neurons.^484^ Further, GPR39 increases Na^+^/H^+^ exchanger activity in hippocampal neurons in a pH-dependent process.^485^

#### GPR40

GPR40 (also known as free fatty acid receptor 1) is the receptor for medium and long-chain unsaturated fatty acids. GPR40 activates the NOD-like receptor pyrin domain-containing protein 3 (NLRP3) inflammasome pathway by blocking the formation of apoptosis-associated speck-like protein containing a CARD (an inflammasome component).^486^ GPR40 promotes hypothalamic neurogenesis by enhancing cell proliferation and survival.^487^ GPR40 may associate with the development of epilepsy by altering N-methyl-d-aspartate receptor-mediated synaptic transmission.^488^ In Alzheimer’s disease model, activating the GPR40 receptor can reduce β-amyloid production and rescue cognitive deficits.^489,490^

#### GPR50

GPR50 exhibits high sequence homology with melatonin MT1/2 receptors. However, melatonin (the endogenous ligand for MT1/2 receptors) cannot bind to GPR50 directly.^491^ GPR50 can be detected in the pituitary, hypothalamus, and hippocampus intermedia.^491,492^ GPR50 enhances neuronal differentiation via notch and WNT/β-catenin.^493^ GPR50 might be involved in psychiatric illness by interacting with neurite outgrowth inhibitor NOGO-A.^494^ GPR50 is an X-linked gene (Xq28). It is suggested to be a sex-specific risk factor in bipolar affective disorder, major depressive disorder, and schizophrenia.^495^ GPR50 can antagonize the MT1 receptor by forming a heterodimer.^496^ The inhibitory effects are mediated via the large C-terminal tail, which blocks the β-arrestin recruitment and G protein coupling.^495^ MT2 receptor could also form a heterodimer with GPR50, but the functional consequence remains to be defined.^496^

#### GPR52

GPR52, a striatal-enriched orphan GPCR. GPR52 stabilizes HTT by cAMP-dependent but PKA-independent mechanisms.^497^ GPR52 antagonist can ameliorate Huntington disease-like phenotypes by diminishing mHTT protein levels.^498^ GPR52 is a potential target of antipsychotic drugs.^499^ GPR52 is associated with cognitive function, emotion, and psychosis-related/antipsychotic-like behaviors.^204,499,500^ GPR52 has high sequence homology with histamine H2 receptor and 5-HT4 receptor.^204^ GPR52 agonist treatment suppresses methamphetamine-induced hyperactivity suggesting that GPR52 might be involved in neurochemical sensitization.^501^ Recent study reveals that GPR52 is a self-activating receptor.^502^ The extracellular loop 2 is immersed deeply into the typical ligand binding pocket of GPR52, which maintains the constitutive active state at physiological conditions.^503^

#### Super-conserved receptors expressed in the brain

GPR27, GPR85, and GPR173 are super-conserved receptors expressed in the brain (SREB). GRR27 deletion is associated with speech delay, contractures, hypertonia, and blepharophimosis.^504^ GPR85 may function as a negative regulator in hippocampal adult neurogenesis and alters cognitive functions, including learning and memory.^505^ It has been reported that GPR85 is a risk factor for schizophrenia.^505^ GPR173 may function by interacting with phoenixin (a recently discovered peptide controlling reproductive hormone secretion, visceral pain, and pruritus) in hypothalamic neurons, which regulates memory and anxiety.^506,507^ In neuronal M17 cells, phoenixin promotes neuronal mitochondrial activity and biogenesis by activating the CREB pathway.^508^ Further, binding of gonadotropin-releasing hormone 1–5 (GnRH 1–5) to GPR173 could inhibit neuronal migration.^509^

#### GPR88

GPR88 expresses exclusively in the neuron of the rat brain throughout the striatum.^510^ In GABAergic medium spiny neurons (MSNs), GPR88 contributes to tonic GABAergic inhibition and responses to GABA release.^511^ GPR88 might play a part in prepulse inhibition of startle, apomorphine-induced climbing, and amphetamine-stimulated locomotor activity.^512^ Co-expression of GPR88 and D1 dopamine receptors is found in the brain.^513^ In Parkinson’s disease (unilateral 6-hydroxydopamine-lesioned rats), GPR88 expression is associated with L-DOPA-mediated behavioural changes.^510^ Antidepressant treatments can modulate GPR88 expression in rat brains.^514^ Morphine can regulate GPR88 expression in the amygdala via the mu-opioid receptor.^515^ GPR88 is genetically associated with various neuropsychiatric disorders, including schizophrenia, bipolar disorder, speech delay, and chorea.^516,517^

### Class B1 (secretin)

#### Structural highlights

**Fig. 7:**
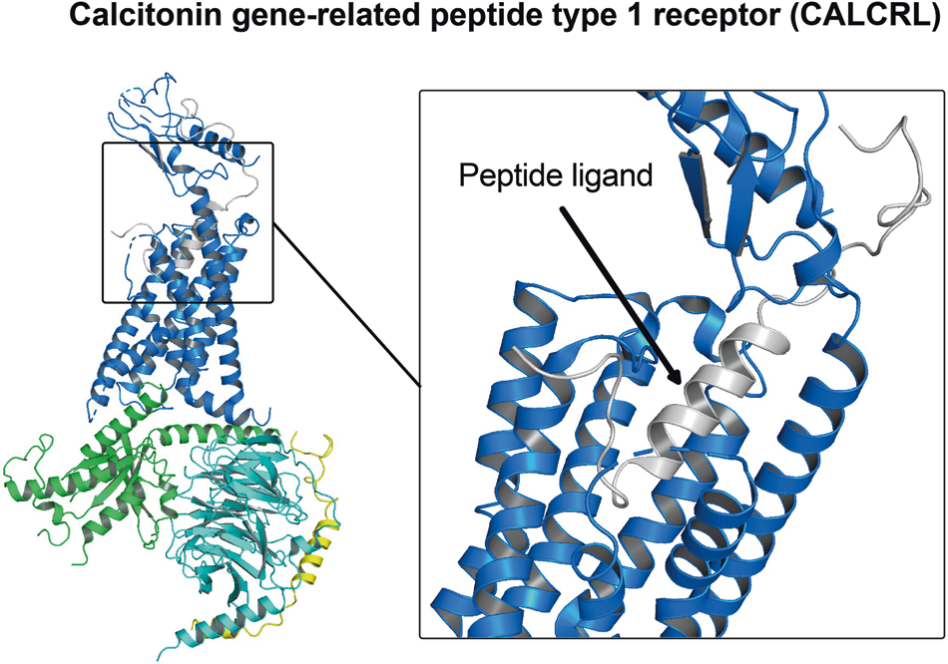
Structural features of class B1 GPCR. Binding of peptide ligand activates calcitonin receptor-like receptor (PDB 6UVA)

Class B1 GPCRs have a conserved extracellular N-terminal domain (ECD) with a three-layered α-β-β/α fold structure (100 to 160 residues) responsible for the binding of peptide hormones (Fig. 7).^518–520^ Peptide ligands stabilize receptors by interacting with both ECD and transmembrane core.^521^ N-terminus of the peptide interacts with the orthosteric pocket within the transmembrane domain.^522,523^ Class B1 GPCRs recognize peptide ligands with different C-terminus, ranging from disordered secondary structures to continuous α-helix.^524,525^ Like class A GPCRs, the cavity formed by the receptor cytoplasmic part allows anchoring of the α5 helix of G proteins.^526,527^ Among class B1 GPCRs, calcitonin and calcitonin gene-related peptide receptors, corticotropin-releasing factor receptors, and the glucagon receptor family are frequently reported to be involved in neurodegenerative diseases and psychiatric disorders.

#### Receptors for calcitonin and calcitonin gene-related peptides

Calcitonin (CT) and calcitonin gene-related peptides (CGRPs) are ligands of the CT receptor. CGRPs also exert their biological functions through CL (calcitonin receptor-like) receptors.^528^

The activity of CT and CL receptors is modulated by receptor activity-modifying protein (RAMP_1-3_).^529^ CT receptor-RAMP complexes can also interact with amylin. Therefore they are also known as amylin receptors (AMY_1-3_).^529^ CT receptors are implicated in neuroinflammation in Alzheimer’s disease.^530^ Antagonists targeting amylin receptors might be beneficial for Alzheimer’s disease treatment.^531^

#### Corticotropin-releasing factor receptor

Corticotropin-releasing hormone (CRF) regulates the neuroendocrine stress response.^532^ CRH exerts its biological function through two receptors: CRFR1 and CRFR2. Human corticotropin-releasing factor receptor 1 **(**CRFR1) exhibits widespread distribution in the central nervous system. In contrast, human CRFR2 is predominately expressed in peripheral tissues.^532^ CRFR1 signaling shows sex divergence in Alzheimer’s disease.^533^ CRFR1 antagonist treatment delays Alzheimer’s disease symptoms, including cognitive impairment and accumulation of Aβ amyloid plaques, by regulating oxidative stress in transgenic mice.^534^ CRF/CRFR1 signaling plays a crucial role in stress-induced behaviour.^532^ It has been shown that noise exposure can increase CRF/CRFR1 expression in the hippocampus.^535^ CRFR1 could sensitize 5-HT2 receptor signaling to modulate anxiety behavior.^536^ In addition, CRFR1 antagonist modulates gamma-aminobutyric acid (GABA)-ergic activity in the brain and controls fear response in rat anxiety models.^537^ Single-nucleotide polymorphisms of CRFR1/2 are positively associated with major depressive disorder.^538–540^

#### Glucagon receptor family

The glucose-dependent insulinotropic polypeptide (GIP) and glucagon-like peptide-1/2 (GLP-1/2) are gut peptide hormones.^541^ The hormones can pass through the blood-brain barrier.^542^ GIP and GIP receptors are expressed throughout the central nervous system.^543,544^ Protease-resistant analog of GIP is designed to treat type 2 diabetes mellitus by controlling weight and improving glycaemic control.^545,546^ Clinical trials indicate that GIP and GLP-1 analogs exhibit therapeutic effects for neurodegenerative diseases.^547^ GLP-1 enhances the supportive function of astrocytes to neurons.^548^ Activated GLP-2 receptor protects hippocampal cells from glutamate-induced cell death and increases the growth of astrocytes.^549^ GLP-1 mimetic reduces oxidative stress and inflammation and promotes neuron formation.^550,551^ GIP can alleviate amyloid beta-induced toxicity in Alzheimer’s disease and relieve symptoms of Parkinson’s disease.^541,542^

### Class B2 (adhesion)

#### Structural highlights

**Fig. 8:**
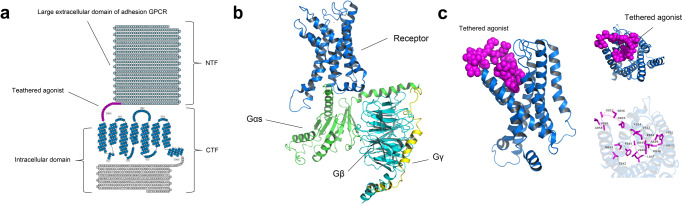
Structural features of class B2 adhesion GPCR ADGRL3 (PDB 7SF7). **a** Schematic representation of ADGRL3 showing characteristic large N-termini. Source: https://gpcrdb.org/protein/agrl3_human/. **b** GPCR of adhesion GPCR coupled to Gαs protein after activation by tethered agonist. **c** Tethered agonist (TA) indicated in spheres. TA occupies the orthosteric pocket of ADGRL3 which as self-agonist for receptor activation

Class B2 GPCR, also known as adhesion GPCR, has a large extracellular domain (ECD). ECD is responsible for the adhesive function exhibiting high structural diversity (Fig. 8a, b).^552^ Adhesion GPCRs are essential for the early development of the nervous system and the brain.^553^ The receptor allows neural cells to communicate with the surrounding environment and migrate to destinate sites to carry out specific functions.^554^ In mouse Purkinje neurons, adhesion GPCR is required to generate intricate dendritic structures for synaptic connections.^554^ Adhesion GPCRs are further classified into ADGRL, ADGRE, ADGRA, ADGRC, ADGRD, ADGRF, ADGRB, ADGRG, and ADGRV subfamilies.^555^

Nearly all class B2 orthologs have the GPCR autoproteolysis inducing domain (GAIN). The GAIN domain is located at the juxtamembrane region.^556^ GAIN domain is crucial for the maturation and function of adhesion GPCR. GAIN possesses intrinsic autoproteolytic activity and cleaves at the integral cysteine-rich GPCR proteolysis site (GPS).^556^ Autoproteolysis give rise to two noncovalently associated fragments: N-terminal fragment (NTF) with most of the extracellular domain; and C-terminal fragment (CTF) consisting of a small proportion of the GAIN domain and most of the entire transmembrane domain (Fig. 8a).^554,557,558^

The activation mechanism of adhesion GPCR is the least understood among different GPCR classes. Most adhesion GPCRs are orphan GPCRs as their natural ligands remain poorly defined.^552^ Receptor activation may follow the tethered-peptide-agonist models.^558^ The stalk region bends approximately 180º downward into the core of the 7TM domain, which functions as tethered agonist to initiate G protein signaling (Fig. 8c).^559,560^ Cleavage-independent mechanisms may exist for receptor activation.^560^ Ligand binding at the GAIN domain might induce conformational changes, which initiate transient G protein signaling.^561^ Upon activation, the intracellular milieu is in the open conformation facilitating G protein coupling. Adhesion GPCRs could employ non–G protein such as PDZ/SH3 domain-proteins and arrestins for signal transduction.^562^

#### Examples of class B2 GPCR

Adhesion G protein-coupled receptor B1 (ADGRB1 or brain-specific angiogenesis inhibitor 1, BAI1) regulates synaptic plasticity in learning and memory processes in the hippocampus.^563^ ADGRB1 is a post-synaptic receptor controlling excitatory synapse development.^564,565^ Forced ADGRB1 attenuates toxin-induced neuronal cell death.^566^ ADGRB1 is associated with dopaminergic neuronal loss in Parkinson’s disease.^566^

Adhesion G protein-coupled receptor B3 (ADGRB3) is enriched in post-synaptic density and cerebellar Purkinje cells.^563,567,568^ ADGRB3 modulates synaptic connection in the cerebellum.^568^ SNPs and gene amplification in ADGRB3 are associated with familial schizophrenia.^569^ Other psychiatric conditions, such as bipolar disorder, are suggested to be linked with ADGRB3.^570^

Adhesion G protein-coupled receptor L3 (ADGRL3) is genetically associated with attention deficit/hyperactivity disorder (ADHD) in adults.^571^ Knockout mice models show enhanced locomotive activity, improved levels of impulsivity, and working memory deficits.^572^ Maternal smoking during pregnancy is an environmental risk factor for ADHD.^573^ In fibroblast cells, nicotine exposure could stimulate ADGRL3 expression.^571^ The downstream ADGRL3 signaling events leading to ADHD remains poorly defined.^574^ ADGRL3 might alter monoaminergic signaling by modulating the expression of dopamine and serotonin transporters.^575^

### Class C (glutamate)

#### Calcium-sensing receptor

Calcium-sensing (CaS) receptor participates in the regulation of Ca^2+^ homeostasis. In Alzheimer’s disease model, elevated expression of CaS receptor is observed in the hippocampal CA1 area and dentate gyrus, which is in accord with the β-amyloid plaques increase.^576^ CaS receptor impeding amyloid-β42 oligomers (Aβ42-os) proteolysis via direct interaction, leading to Aβ42-os aggregation and oversecretion.^577^ CaS receptor inhibitor sustains mental competence by promoting Aβ42 proteolysis.^577^ Inhibiting the CaS receptor improves memory and cognitive defects caused by β-amyloid in mice.^578^ CaS receptor might induce cognitive defects via eliciting cytosolic phospholipase A2 and prostaglandin E2 signaling pathway.^578^

### Class F

#### Structural highlights of Class F GPCR

**Fig. 9:**
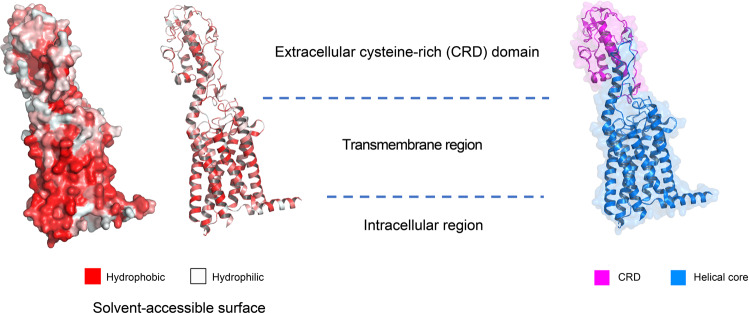
Structural features of class F GPCR. Smoothened homolog SMO (PDB 5L7D) with large extracellular and cysteine-rich (CRD) domain. Solvent-accessible surface. Hydrophobic surface (red); hydrophilic surface (white)

Class F GPCR contains a large extracellular and cysteine-rich (CRD) domain (Fig. 9).^579^ CRD is essential for the stability and activity of class F GPCRs.^580^ FZD gene family is highly conserved in mammals with conserved structural features. FZD is a receptor for the WNT family of lipoglycoprotein, which mediates signal transduction via canonical WNT-β-catenin pathway and β-catenin-independent noncanonical pathways. The secretory WNT binds to the cysteine-rich domain at the extracellular side. The Lys-Thr-X-X-X-Trp (KTXXXW) motif located at the C-terminal is essential for activating the canonical WNT/ β-catenin pathway.^581,582^ WNT signaling regulates neuronal polarization and axon specification polarity by activating atypical protein kinase C in rat hippocampal neurons.^583^ Further, WNT signaling governs collateral or terminal branching of the axon, dendrite outgrowth and guidance, dendritic spine formation, synapse formation/plasticity, and elimination.^584^ WNT/FZD signaling alterations are observed in several neurological disorders, including Alzheimer’s disease and Huntington’s disease.^585,586^ The transmembrane region is compact and hydrophilic.^580,587^ Similar to class A GPCR, outward bending of TM6 and an inward shift of TM5 at the cytoplasmic side is observed in the active class F GPCR.^580^

Class F receptors frizzled (FZD1-10) and smoothened (SMO) are closely associated with embryonic development and tissue homeostasis.^588^ Reported FZD ligands include frizzled-related proteins (SFRPs) and R-spondin.^589,590^ FZD1 is found in dopamine-synthesizing neurons, which form an astrocyte-DA autoprotective loop via WNT1/FZD1/β-catenin signaling.^591^ FZD1 enhances myelin preservation and neuronal survival;^592^ FZD3 is genetically related to substance-induced psychosis and schizophrenia;^593,594^ Neuronal degeneration observed in amyotrophic lateral sclerosis is regulated by WNT5a/FZD4 signaling.^595^ WNT5a/FZD5 activity is associated with neuronal inflammatory signaling;^596^ Genetic FZD6 variants are associated with neural tube defects in the central nervous system;^597^ FZD9 deletion is noted in patients with Williams-Beuren syndrome, a rare genetic disorder with mild to moderate intellectual disability or learning difficulties^598^ FZD10 may play a role in brain vascular development;^599^ SMO is the receptor for hedgehog proteins involved in neuronal/ glial proliferation and tissue regeneration.^600^

## Concluding remarks

GPCRs are cooperatively involved in the manifestations of neuropsychiatric disorders. Elucidating the intrinsic signaling preference of G proteins or arrestins helps to improve drug efficacy and side-effect profiles. GPCR can work in the dimeric form in disease development. Characterizing the allosteric interactions and the functional consequences of GPCR dimers might provide insights into the pathogenesis of neuropsychiatric disorders. Apart from acting directly in the nervous system, GPCRs might contribute to disease development via the immune system.^220^

Target identification is challenging as the clinical presentations are resulted from heterogeneous biological, genetic, and environmental factors. Nevertheless, the increasing understanding of GPCR functions opens a new possibility in drug discovery. Most of the drugs targeting GPCR lack subtype-selectivity.^601^ Local drug administration may require to avoid debilitating side effects.^602^ The development of psychiatric medications remains slow as the pharmaceutical industry pays more attention to antidepressants and antipsychotic drug development.^603^ Therefore, developing specific therapeutic modulators which could recognize subtypes with high specificity is crucial for effective drug development.^602^

Benefiting from the advances in crystallography and cryo-electron microscopy technology, the resolved GPCR structures increase our understanding of GPCR functions in pathological conditions. Detailed protein structures could reveal crucial ligand binding features in physiological conditions.^215,547,604^ Detailed receptor/ligand profile could facilitate lead compound identification and drug optimization. Hence, harnessing our knowledge of molecular mechanisms and structural information of GPCR will be advantageous for developing effective treatments against neuropsychiatric disorders.
